# Hypoxia‐induced secretory autophagy in cancer‐associated fibroblasts promotes ECM remodelling through serglycin secretion in oral squamous cell carcinoma

**DOI:** 10.1002/ctm2.70556

**Published:** 2025-12-18

**Authors:** Yan Zhang, Cheng Tao, Xiteng Yin, Zhi Wang, Yuyang Zhang, Jiale Yu, Yufeng Wang, Wei Han

**Affiliations:** ^1^ Department of Oral and Maxillofacial Surgery Nanjing Stomatological Hospital Affiliated Hospital of Medical School Institute of Stomatology Nanjing University Jiangsu China

**Keywords:** cancer‐associated fibroblasts, ECM remodelling, secretory autophagy, serglycin

## Abstract

**Background:**

The worst pattern of invasion (WPOI) is a critical histological prognostic indicator in oral squamous cell carcinoma (OSCC), yet the underlying mechanisms driving high WPOI remain poorly understood. While cancer‐associated fibroblasts (CAFs) and their secreted factor serglycin (SRGN) are implicated in tumour progression, the regulation of SRGN secretion within the hypoxic tumour microenvironment is unknown.

**Methods:**

We performed single‐cell RNA sequencing (scRNA‐seq) on 6 OSCC samples (3 each of WPOI 1–3 and 4–5) to identify subgroups of CAFs and their characteristic gene expression profiles. Using Western blot, qRT‐PCR, and immunofluorescence, we investigated hypoxia‐induced SRGN secretion pathways. Complementary CRISPR‐Cas9 knockout, Co‐IP assays, and xenograft models elucidated SRGN's role in ECM remodelling.

**Results:**

ScRNA‐seq revealed significant enrichment of CAFs, particularly an SRGN‐expressing myCAF subpopulation, in high‐WPOI (4–5) OSCC tissues. Under hypoxia, CAFs switched SRGN secretion from the conventional ER‐Golgi pathway to an unconventional secretory autophagy pathway, dependent on autophagosome formation but independent of lysosomal degradation. Secreted SRGN directly interacted with matrix metalloproteinase 2 (MMP2) and matrix metalloproteinase 9 (MMP9) in the extracellular matrix (ECM), enhancing ECM remodelling and OSCC invasion and migration. In vivo, either genetic ablation of SRGN in CAFs or pharmacological inhibition of autophagy significantly suppressed tumour growth, inhibited collagen I degradation, and restored E‐cadherin expression.

**Conclusion:**

Our study identifies a novel mechanism whereby hypoxia induces CAFs to secrete SRGN via secretory autophagy. This SRGN‐MMP2/9 axis drives ECM remodelling and promotes OSCC invasion, which histologically manifests as high WPOI. Targeting secretory autophagy or SRGN represents a promising therapeutic strategy for aggressive OSCC.

**Key points:**

1. Under normoxia, SRGN enters the conventional secretory pathway via the endoplasmic reticulum (ER) and Golgi apparatus for extracellular release.
2. Under hypoxia, elevated autophagy levels in CAFs facilitate the release of SRGN into the ECM through secretory autophagy‐mediated plasma membrane fusion.
3. Within the ECM, SRGN interacts with MMP2 and MMP9, enhancing ECM remodelling and OSCC invasion.

## Graphical abstract

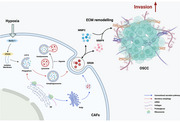

## 1 INTRODUCTION

Oral squamous cell carcinoma (OSCC), the predominant form of head and neck cancer, represented approximately 2% of newly diagnosed cancer cases and was responsible for 1.8% of cancer‐related fatalities in 2020.^1^ Brandwein‐Gensler et al. established a risk‐stratification model for OSCC prognosis, including the pattern of infiltration (POI)—a histological measure of tumour invasion at the host‐tumour margin.^2^ There are five types of POI, the first three being low infiltrative, while the last two have a more diffuse infiltration pattern, which also represents a higher degree of infiltration.^2^ The worst pattern of invasion (WPOI) is defined as the highest score for each patient, representing the most reliable prognostic indicator for early‐stage OSCC.^3^, ^4^, ^5^, ^6^

The tumour microenvironment (TME), which encompasses the surrounding area where tumour cells grow and evolve, shows unique patterns of remodelling in OSCC cases with different WPOI grades.^7^ In the TME, unfavourable physiological conditions such as hypoxia can induce autophagy in CAFs, which may lead to tumorigenesis, invasion, metastasis, and therapy resistance.^8^, ^9^, ^10^ Autophagy is an evolutionarily conserved cellular process in which intracellular components (e.g., proteins, organelles, and pathogens) are delivered to lysosomes for degradation and recycling—a process known as degradative autophagy.^11^ Recent studies have shown that autophagy also contributes to protein secretion. This process, known as secretory autophagy, represents a key unconventional secretory pathway.^12^

Serglycin (SRGN) is a secretory granule proteoglycan that binds and regulates bioactive molecules such as cytokines and proteases through its glycosaminoglycan (GAG) chains, playing a central role in inflammation, immune modulation, and TME regulation.^13^ In the TME, SRGN supports tumour growth, invasion, and metastasis through mechanisms that affect immune cell function, promote angiogenesis, remodel the extracellular matrix, and facilitate immune evasion.^13^, ^14^, ^15^ Interestingly, SRGN, a secretory factor produced by CAFs, has been reported to be highly expressed in various cancers—including breast, lung, nasopharyngeal, and oesophageal—and is associated with promoting cancer invasion, metastasis, and chemoresistance.^15^, ^16^, ^17^, ^18^, ^19^ It has been reported in the literature that SRGN is a downstream target gene of HIF‐1α, and abnormal HIF‐1α/SRGN regulation affects tumour progression and metastasis.^20^ We have previously demonstrated that hypoxia can stimulate CAFs to secrete SRGN by activating the Wnt/β‐catenin pathway, thereby promoting head and neck squamous carcinoma (HNSCC) tumour cell growth.^21^ However, the specific mechanism regarding the secretion of SRGN by CAFs stimulated by hypoxia is still unclear.

In this study, single‐cell RNA sequencing (scRNA‐seq) analysis revealed significant enrichment of CAFs in high‐grade (WPOI 4–5) OSCC tissues, with elevated SRGN expression in myCAFs potentially correlating with WPOI classification. Further investigation demonstrated that under hypoxic conditions, CAFs preferentially secrete SRGN into the ECM via secretory autophagy. The released SRGN promotes ECM remodelling through direct interactions with matrix metalloproteinase 2 (MMP2) and matrix metalloproteinase 9 (MMP9), thereby enhancing the invasive and migratory capacities of OSCC cells.

## 2 MATERIALS AND METHODS

### 2.1 Cells and culture conditions

The human tongue squamous cell carcinoma lines SCC9, CAL27, and HN6 (from the Ninth Hospital of Shanghai) were grown in high‐glucose DMEM complete medium (KeyGEN BioTECH, China) with 10% FBS, at 37°C and 5% CO_2_. Concurrently, CAFs were maintained in Fibroblast Medium (FM; ScienCell) supplemented with 5% FBS and fibroblast growth supplements. To investigate hypoxic effects, CAFs, HN6, SCC9, and CAL27 cells were cultured for 24 hours under either 20% or 1% oxygen conditions.

### 2.2 Patients and tissue samples

Primary CAFs were isolated from 20 fresh OSCC tumour samples. Tissues were washed with PBS containing 20% penicillin‐streptomycin, minced into 1 mm^3^ fragments, and cultured in 10 cm^2^ dishes (inverted for 4 h, then maintained undisturbed for 4 days). After medium replacement, outgrown fibroblasts were expanded (passages 4–8) and validated as CAFs by positive staining for α‐SMA/FAP and negative staining for E‐cadherin. The study was approved by the Nanjing Stomatological Hospital Ethics Committee.

### 2.3 Single‐cell RNA‐seq data preprocessing

Fresh OSCC tissue samples (three WPOI 1–3 and three WPOI 4–5) were processed within 1 h post‐surgery. Tissues were enzymatically dissociated and filtered to obtain single‐cell suspensions (> 90% viability). scRNA‐seq data were analyzed using Seurat 4.0.0, with quality control filtering cells based on gene and UMI count, and removing doublets using DoubletFinder. After normalization and batch correction, we performed clustering and UMAP visualization, identifying marker genes (|log2FC| > .58, *p* < .05). Functional enrichment analysis was done using R 4.0.3 with support from OE Biotech Co., Ltd. (Shanghai, China).

### 2.4 Measurement of tissue Young's modulus by SICM

Young's modulus mapping was performed on a tissue section using a laboratory‐assembled scanning ion‐conductance microscope (SICM).^22^ Tissue section on a standard Petri dish was immersed in.01 M phosphate‐buffered saline (PBS) at room temperature. A borosilicate nanopipette (≈250 nm inner tip) filled with PBS was pressurized to 15 kPa, providing a steady hydrojet. The Z position corresponding to a 1% drop in current marked the sample topography, while the additional approach needed to reach a 2% reduction provided the indentation depth. Young's modulus was then calculated with the Rheinlaender–Schäffer algorithm.^23^ Data handling and statistical tests were carried out in MATLAB R2024b (MathWorks), and graphs were prepared in Origin 2023b (OriginLab).

### 2.5 RNA extraction and qRT‐PCR

RNA was extracted using the SteadyPure RNA extraction kit (ACCURATE BIOTECHNOLOGY(HUNAN) CO., LTD, Changsha, China). The purity and concentration of the isolated RNA were assessed with a NanoDrop One spectrophotometer according to the manufacturer's guidelines. First‐strand cDNA was synthesized using HiScript II Q RT SuperMix. Quantitative PCR was performed on a Step‐One Plus system using ChamQ Universal SYBR qPCR Master Mix (Vazyme Biotech, China), with β‐actin serving as the internal control. Gene expression levels were determined via the 2^−ΔΔCt^ method. Sequences for siRNAs: si‐NC, UUCUCCGAACGUGUCACGUTT; si‐ATG5#1, GGAUGCAAUUGAAGCUCAUTT; si‐ATG5#2, CCAAGAGUAAGUUAUUUGATT; si‐ATG5#3, CGCUAUAUCAGGAUGAGAUTT; si‐Beclin 1#1, GCUGCCGUUAUACUGUUCUTT; si‐Beclin 1#2, GGAUGACAGUGAACAGUUATT; si‐Beclin 1#3, CUGGACACGAGUUUCAAGATT.

### 2.6 Immunohistochemical assay

Immunohistochemical (IHC) detection was conducted following standard processing of 4 µm FFPE sections through deparaffinization and rehydration, antigen retrieval and peroxidase blocking were implemented. Overnight incubation at 4°C with primary antibodies (SRGN, A6951, 1:100, ABclonal, China; COL1A1, A1352, 1:200, ABclonal; E‐cadherin, 20874‐1‐AP, 1:5000, Proteintech, USA; MMP2, 10373‐2‐AP, 1:100, Proteintech; MMP9, 10375‐2‐AP, 1:50, Proteintech; HIF1α, PB9253, 1:100, Boster, China) at 4°C overnight. Subsequent processing included 10‐minute incubation with secondary antibody at RT, DAB chromogenic development, and hematoxylin counterstaining.

### 2.7 Knockout of SRGN by CRISPR‐Cas9

CRISPR‐Cas9‐mediated SRGN knockout (SRGN KO) was performed using commercially obtained RNP complexes (Haixing Bioscience, China) containing hSpCas9 and designed gRNAs (targeting exons 2–3: GCAGGGTCGATCGCATTCTGAGG and GCACTAGGGTGTGATGTACTAGG; designed via CRISPR MIT). Cellular delivery was accomplished through electroporation (Neon system, ThermoFisher, USA). Following 48 h of recovery, single‐cell colonies were isolated and expanded. Target site analysis involved genomic DNA extraction (Zymo Research, USA), PCR amplification (Vazyme P112 Master Mix, China), and sequencing verification (GENEWIZ). Established cell lines with various genotypes (+/+, +/−, +/−A, +/−B, −/−) were cultured under identical conditions to parental cells.

### 2.8 Supernatant collection and ELISA

Conditioned media were harvested from CAFs grown in 6‐well plates (6 × 10⁵ cells/well) following centrifugation (1000 rpm, 10 min). SRGN secretion levels were quantified using a commercial human SRGN ELISA kit (Cloud‐Clone Corp, USA) according to the manufacturer's protocol. Sample concentrations were calculated by plotting OD values against the standard curve.

### 2.9 Protein extraction and western blot

Cells (1 × 10⁵/well) were seeded in 6‐well plates for 24 h. Proteins were extracted with RIPA buffer (Beyotime P0013B, China) and quantified by BCA assay (Beyotime P0009). After SDS‐PAGE (4‐20%) and PVDF transfer, membranes were blocked with 5% milk (2 h), incubated with primary antibodies (4°C, overnight) and secondary antibodies, then visualized by ECL. Quantification used ImageJ. The primary antibodies used are listed as follows: SRGN (1:1000, A25426, ABclonal), ATG5 (1:1000, 10181‐2‐AP, Proteintech), LC3B (1:2000, ab192890, Abcam, USA), Beclin 1 (1:1000, 11306‐1‐AP, Proteintech), β‐actin (1:4000, 20536‐1‐AP, Proteintech), LAMP1 (1:4000, 21997‐1‐AP, Proteintech), P62 (1:4000, 18420‐1‐AP, Proteintech), CTSD (1:4000, 21327‐1‐AP, Proteintech), MMP9 (1:1000, bs‐4593R, Bioss, China), MMP2 (1:1000, CY5189, Abways, China), and MMP11 (1:1000, CY5778, Abways).

### 2.10 Co‐immunoprecipitation and immunoprecipitation

Co‐immunoprecipitation (Co‐IP) and immunoprecipitation (IP) were performed using the IP/Co‐IP kit (ThermoFisher Scientific, 88805) following to the manufacturer's instructions. Primary antibodies used in this assay included: β‐actin (1:4000, 20536‐1, Proteintech), MMP9 (1:1000, bs‐4593R, Bioss), MMP2 (1:1000, CY5189, Abways), MMP11 (1:1000, CY5778, Abways), Flag (1:5000, 80801‐2‐RR, Proteintech).

### 2.11 Immunofluorescence assay

Cells were fixed, permeabilized, and blocked sequentially using 4% PFA (20 min),.5% Triton X‐100 (10 min, 4°C), and a solution of 5% goat serum with 1% BSA (30 min). After overnight primary antibody incubation (4°C), fluorophore‐conjugated secondary antibodies were applied (1 h, RT). Nuclei were counterstained with DAPI (10 min), and images were acquired by confocal microscopy. Lysosomal detection was accomplished using LysoSensorTM Green DND‐189 (ThermoFisher A66436) with immediate image acquisition. The following primary antibodies are used: SRGN (1:200, sc‐374657, Santa Cruz, Bolivia), LC3B (1:200, ab192890, Abcam), LAMP1(1:4000, 21997‐1‐AP, Proteintech), GM130(1:200, 11308‐1‐AP, Proteintech).

### 2.12 Transmission electron microscopy

The samples were fixed with 1% osmium tetroxide for 1–2 h after initial fixation and phosphate buffer rinses, then dehydrated through a graded ethanol series (30% to 100%) followed by acetone. For embedding, the samples were gradually infiltrated with embedding resin through increasing resin/acetone ratios (1:1 for 1 h, 3:1 for 3 h) before final pure resin infiltration overnight. After polymerization at 70°C, ultrathin sections (70‐90 nm) were cut and stained with lead citrate and uranyl acetate prior to transmission electron microscopy (TEM) observation.

### 2.13 Transwell invasion assay

Invasion assays were performed using transwell chambers (8.0 µm pores; Corning, USA) pre‐coated with Matrigel matrix. After 1 h Matrigel polymerization (37°C, 5% CO_2_), inserts were rehydrated with serum‐free DMEM (100 µL). The upper chamber was inoculated with 2 × 10⁵ cells suspended in low‐serum medium (.5‐1% FBS), while the lower compartment contained CAF‐derived conditioned medium. After 24 h incubation, invasion cells were fixed and stained with.4% crystal violet. Quantitative analysis involved counting cells in five random microscopic fields per chamber (triplicate samples; mean ± SD).

### 2.14 Wound healing assay

SCC9 cells were seeded in six‐well plates (2 × 10⁵ cells/well) and cultured to confluence in DMEM with 10% FBS (37°C, 5% CO_2_, 24 h). Uniform wounds were created using 200 µL pipette tips, followed by PBS washing and baseline image acquisition. Experimental CAF supernatants (2 mL) were introduced, and cell migration was documented every 12 h during 24 h incubation. The extent of wound closure was determined by the equation: [(initial wound measurement − subsequent measurement)/initial measurement] × 100.

### 2.15 ECM adhesion assay

The 96‐well plates were coated overnight at 4°C with human fibronectin, laminin, or vitronectin and then blocked with 1% BSA for 1 hour at 37°C. Cell suspensions (1 × 10⁴ cells/well) were introduced and permitted to adhere (37°C, 2 h). Following PBS washes, adherent cells were assessed by CCK‐8 assay in serum‐free medium (200 µL/well). The adhesion rate was determined by comparing optical density values using the formula: (experimental OD/control OD) × 100.

### 2.16 ECM degradation assay

Glass‐bottom dishes were coated with Oregon Green 488‐gelatin (ThermoFisher G13187) for 30 min at RT, fixed with 3.6% formaldehyde (15 min), and equilibrated with 10% FBS medium (30 min). CAL27 cells (5 × 10⁴/dish) in conditioned medium were seeded and cultured for 24 h. Cells were then fixed, permeabilized (.5% Triton X‐100), and stained with Phalloidin/DAPI. Confocal microscopy was used to image gelatin degradation (black = degraded; green = intact), quantified in ImageJ by thresholding the degraded/total area ratio.

### 2.17 Xenograft model

A total of twenty‐eight male BALB/C nude mice (6–8 weeks old, male; Jiangsu Huachuang Sino Pharma Tech Co., Ltd, China) were assigned to four groups, each consisting of seven mice: the CAL27 cell group, CAL27+CAFs cell group, CAL27+CAFs (3‐MA) cell group, and CAL27+SRGN KO CAFs cell group. Mice received tongue injections of.2 mL cell suspension (5 × 10⁶ cells/mL). All mice were housed under standard specific pathogen‐free (SPF) conditions with a 12 h light/dark cycle and provided with ad libitum access to food and water. All experimental procedures were conducted in accordance with ethical guidelines to prevent unnecessary suffering. When the following clinical stress symptoms appear, it is considered a humane endpoint: Severe activity limitation: unable to obtain food or water normally; signs of pain: persistent arched back posture, disheveled fur, or vocalization when touched; behavioural changes: severe lethargy, persistent anorexia (no food intake within 24 h), or isolation from cage mates; other critical conditions: difficulty breathing, signs of paralysis, or any other condition indicating severe physiological dysfunction. Tumours were excised and volumes determined by the (width^2^ × length)/2 calculation.

### 2.18 STRING analysis

STRING software (https://string‐db.org/) was used to generate a predicted interaction network for the SRGN protein. This database integrates multiple sources of evidence, including experimental data, genomic features, co‐expression profiles, literature mining, and computational predictions, and employs a confidence scoring system to evaluate protein–protein interactions.

### 2.19 TCGA analysis

This study analyzed data from The Cancer Genome Atlas (TCGA)‐HNSC cohort (https://tcga‐data.nci.nih.gov/tcga/), focusing on OSCC samples with complete clinical and SRGN expression data. To minimize tumour heterogeneity, we calculated a “Stromal Marker Score” based on the expression of 13 stromal genes (such as COL1A1, FN1, and ACTA2) and selected the top 50% of stroma‐rich samples for further analysis. Within this subset, patients were stratified into SRGN‐high and SRGN‐low groups according to median SRGN expression. Kaplan–Meier survival analysis and the log‐rank test were used to compare overall survival between the two groups, with *p*‐values reported to evaluate prognostic significance.

### 2.20 Statistical methods

Statistical evaluations were conducted utilizing GraphPad Prism and ImageJ software, with experimental results expressed as arithmetic mean ± standard deviation. Sample sizes for each condition are detailed in the respective figure captions. Comparisons between groups were conducted using either the Student's *t*‐test or one‐way ANOVA, followed by pairwise *t*‐tests for further analysis. The following probability thresholds were applied: *p* < .05 (*), *p* < .01(**), and *p *< .001(***).

## 3 RESULTS

### 3.1 High WPOI grade tumours exhibit increased CAF infiltration and active ECM remodelling

**FIGURE 1:**
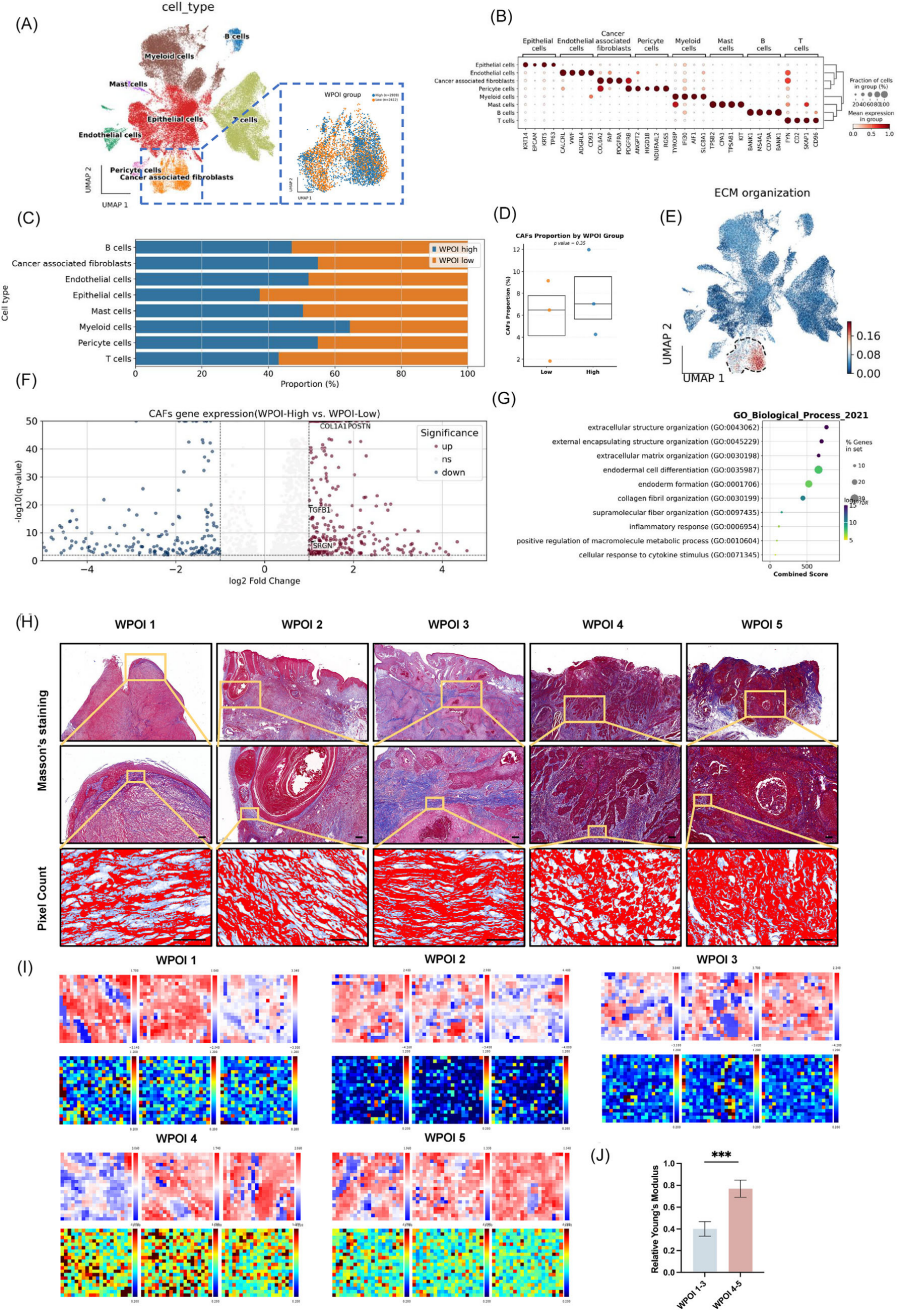
scRNA‐seq revealed CAF‐mediated ECM remodelling and collagen reorganization across WPOI grades in OSCC. (A) UMAP visualization of 8 different cell clusters, colour‐coded by cell type (epithelial cells, CAFs, T cells, etc.). The inset shows the distribution pattern of CAFs based on WPOI. (B) Point plots of marker genes for identified clusters (e.g., KRT5/14 for epithelial cells, PDGFRA for CAFs). (C) Stacked bar graphs showing the proportional changes of major cell clusters in different WPOI classes. (D) The proportion of CAFs in OSCC tissues with different WPOI (WPOI Low vs. WPOI High). (E) Pathway scores associated with ECM remodelling are projected on UMAP space and highlight CAFs cell populations with active ECM remodelling scores. (F) Differential expression analysis of CAFs cell populations after grouping based on WPOI, with red indicating highly expressed genes in high WPOI compared with low WPOI samples, and blue indicating lowly expressed genes, where specific highly expressed genes such as POSTN, COL1A1, TGFB1, and SRGN are labelled. (G) Functional enrichment of differentially expressed genes in CAFs (WPOI4‐5 vs. WPOI1‐3), highlighting ECM organization and remodelling pathways. (H) Histological analysis of OSCC tissues with different WPOI was performed using Masson trichrome staining. Collagen alignment was displayed and quantified for regions of interest using Fiji software. Scale bar = 100 µm. (I, J) The relative values of interstitial stiffness of OSCC tissues with different WPOIs were analyzed by SICM, with three different points measured for each sample (three samples from WPOI 1–3 and 4–5 grades, respectively) (***p < .001).

To investigate the role of CAFs in WPOI formation in OSCC, we performed scRNA‐seq on six fresh OSCC specimens, including three cases each of low‐grade (WPOI 1–3) and high‐grade (WPOI 4–5) tumours. Following rigorous quality control, we obtained high‐quality transcriptomes from 76,832 single cells for subsequent analysis. Our analysis revealed eight distinct cell populations, with CAFs emerging as a prominent cellular component, particularly in high WPOI samples (Figure 1A). Initial data processing included batch effect correction to minimize technical variability between samples (Figure S1A). Cell clustering identified epithelial cells, CAFs, endothelial cells, and other stromal components (Figure 1A). CAFs demonstrated characteristic expression of established markers, including COL6A2, FAP, PDGFRA, and PDGFRB (Figure 1B), with detailed marker expression patterns and UMAP distributions provided in Figure S1E. Comparative analysis of cellular composition revealed distinct patterns between WPOI groups (Figure 1C; Figure S1B,D). Although the Wilcoxon rank‐sum test in Figure 1C did not reach statistical significance (*p* = .35), as shown in Figure 1D, there is a consistent upward trend in the proportion of CAFs in the WPOI 4–5 group. This trend is in line with the biological characteristic reported in previous studies that “CAFs infiltration increases in highly invasive tumours”.^24,25^ Notably, the ECM‐rich pathways exhibit a distinct expression distribution pattern in the UMAP space, with a significant enhancement in the cell cluster region where CAFs are located (Figure 1E). Differential expression analysis further identified upregulation of multiple ECM organization genes in high WPOI CAFs (Figure 1F), with pathway enrichment confirming significant activation of ECM structural organization and collagen metabolic processes in these cells (Figure 1G).

Masson's trichrome staining confirmed increased collagen deposition in high WPOI tissues, with more disorganized fibres compared with low WPOI samples (Figure 1H; Figure S1F). In addition, MMP‐mediated ECM degradation triggers compensatory matrix remodelling—CAFs accelerate the synthesis and crosslinking of newly formed collagen fibres, resulting in a denser and stiffer matrix structure.^26^ SICM measurements also showed higher ECM stiffness in high WPOI tissues (Figure 1I,J). These findings suggest that CAF‐mediated ECM remodelling contributes to invasive patterns in OSCC, especially in high WPOI cases.

### 3.2 High WPOI‐grade OSCC tissues exhibit marked hypoxic features and enrichment of SRGN+ CAFs subpopulation

**FIGURE 2:**
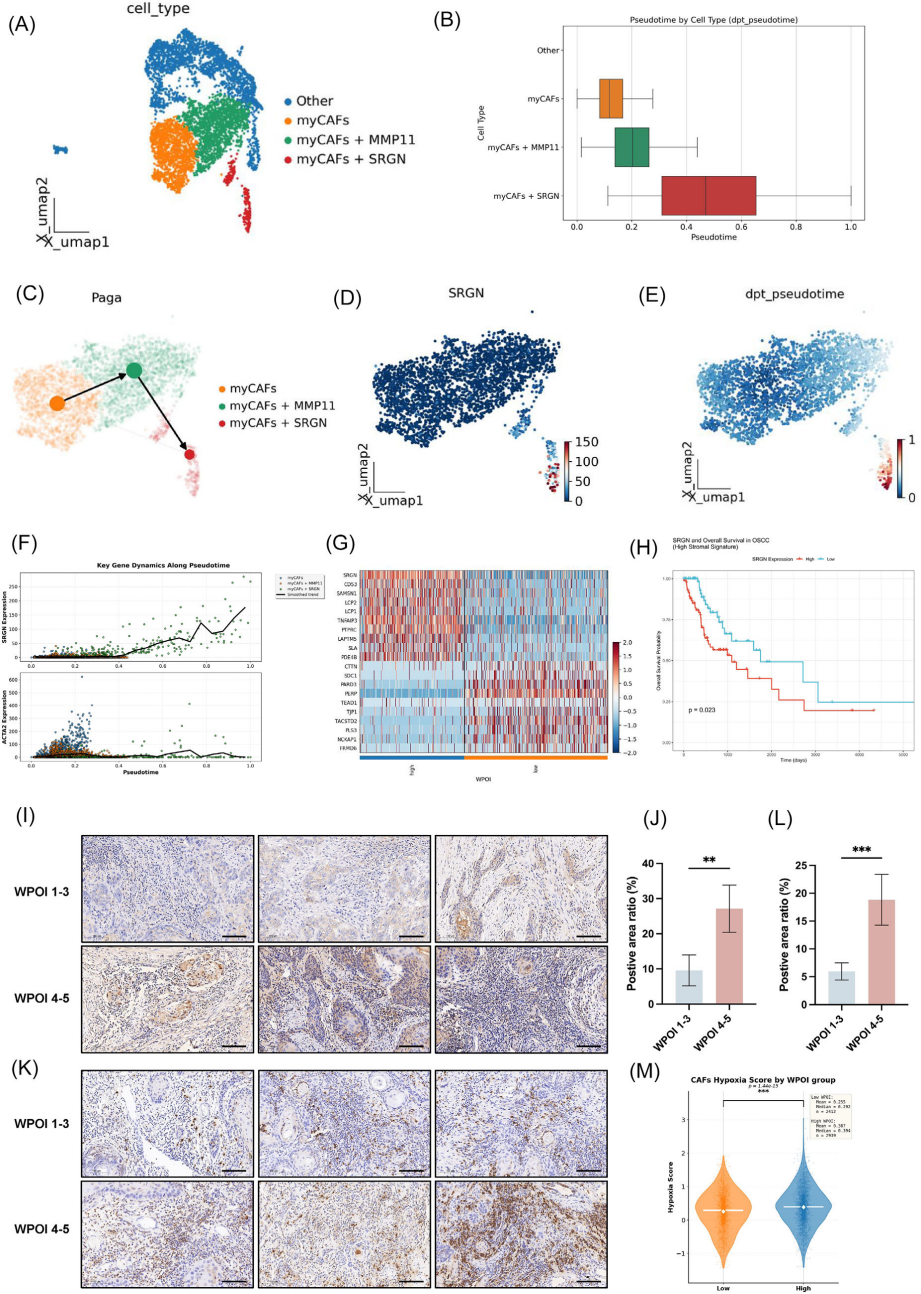
Pseudo‐time analysis reveals dynamic gene expression patterns in myCAF subpopulations and differential SRGN expression across WPOI grades. (A) UMAP visualization of CAF subpopulations, highlighting myCAFs (orange), myCAFs+MMP11 (green) and myCAFs+SRGN (red). (B) Density distribution of pseudo‐temporal values for myCAFs subtypes, indicating time course. (C) Pseudo‐temporal projection on UMAP space showing the developmental trajectory of myCAFs. (D) PAGA trajectory analysis confirming branching events (circle: cell state; edge: connection). (E) SRGN expression in myCAF subpopulations. (F) Dynamic expression profiles of SRGN and ACTA2 (as a comparison) along a pseudo‐temporal sequence showing that SRGN is upregulated in late myCAFs. (G) FoldChange of differentially expressed genes was ranked from largest to smallest, and 20 genes were selected for each of the up‐ and downregulation to draw heat maps. (H) The survival rate of 160 OSCC patients with a high stromal microenvironment from the TCGA database. (I, J) IHC of SRGN protein levels in different WPOI OSCC tissues (*n* = 30). Scale bar = 100 µm (**p < .01). (K, L) IHC of HIF1α protein levels in different WPOI OSCC tissues (*n* = 20). Scale bar = 100 µm (***p < .001). (M) Calculate the CAF hypoxia score for different WPOI OSCC samples using scRNA‐seq data (***p < .001).

Through comprehensive subpopulation analysis of CAFs (Figure 2A), we identified three distinct myofibroblastic CAF (myCAF) subtypes: (1) classical myCAFs (ACTA2+/COL1A1+), (2) MMP11+ myCAFs (MMP11high), associated with ECM remodelling, and (3) SRGN+ myCAFs (SRGNhigh), showing terminal activation markers. Specifically, “other cells”, including proliferative CAFs (proCAFs) and inflammatory CAFs (iCAFs), were also identified (Figure S2A,B). Pseudotime trajectory analysis revealed a unidirectional differentiation from classical myCAFs to SRGN+ myCAFs (Figure 2B,C). This pattern was confirmed by PAGA analysis of branching nodes (Figure 2D). SRGN+ myCAFs had higher gene expression, particularly for ACTA2 (Figure 2E,F). Analysis of differential genes in OSCC samples showed SRGN as a significant factor (Figure 2G). In our previous analysis of 546 HNSCC cases from the TCGA database, we found that high expression of stromal SRGN was significantly associated with poor prognosis.^21^ This study further focuses on 169 OSCC patients with high stromal microenvironment characteristics. After excluding nine cases with low SRGN expression and early death, analysis of the final 160 samples revealed that, in OSCC patients with a high stromal background, those with high SRGN expression had significantly shorter overall survival (log‐rank *p* = .0229) (Figure 2H). IHC of 60 OSCC specimens revealed strong SRGN expression in high‐grade cases, further validating the clinical relevance of SRGN+ CAFs (Figure 2I,J).

Given the critical role of hypoxia in TME, we explored its association with WPOI grading. IHC analysis of HIF‐1α in 40 OSCC tissues showed significantly enhanced signals in the stromal region of high WPOI‐grade samples (Figure 2K,L). Besides, hypoxia scoring of CAFs in different WPOI groups, based on scRNA‐seq, confirmed that CAFs in the WPOI 4–5 group had significantly higher hypoxia scores (*p* < .05, Figure 2M), linking CAF‐specific hypoxia to aggressive tumour invasion patterns.

### 3.3 Hypoxia‐stimulated CAFs secrete SRGN via autophagy

**FIGURE 3:**
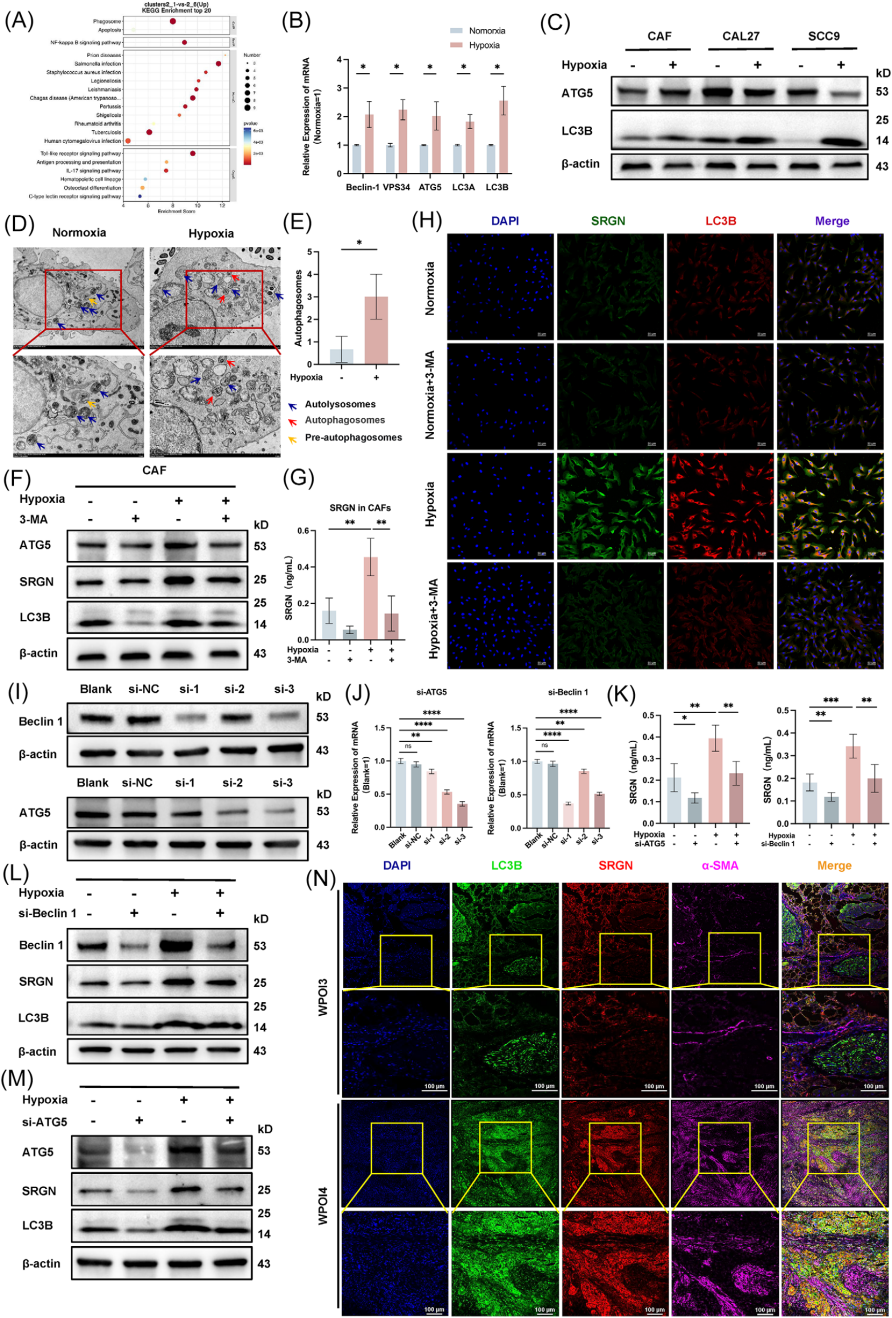
Hypoxia treatment correlates positively with the secretion of SRGN and autophagy levels in CAFs. (A) KEGG enrichment analysis. (B) qPCR analysis of autophagy‐related markers in CAFs under normoxic and hypoxic conditions. (C) WB shows expression levels of ATG5 and LC3B in CAFs, CAL27, and SCC9 cells under normoxic and hypoxic conditions. (D, E) TEM images showing autophagosomes in normoxic and hypoxic CAFs, along with a quantitative analysis of the autophagosomes. Scale bar = 2 µm. (F) WB reveals changes in ATG5, LC3B and SRGN expression in CAFs under normoxic and hypoxic conditions after 3‐MA treatment. (G) ELISA detection of SRGN secretion in the supernatant of normoxic and hypoxic CAFs, with or without 3‐MA treatment. (H) IF observation of co‐expression of SRGN and LC3B in normoxic and hypoxic CAFs with or without 3‐MA. (I, J) qPCR and WB validation of the knockdown efficiency of ATG5 and Beclin 1 in CAFs. (K) ELISA detection of the supernatant from WT CAFs and si‐ATG5/si‐Beclin1 CAFs treated under normoxic and hypoxic conditions for 24 h. (L, M) WB detection of the expression levels of SRGN, LC3B, and ATG5 in WT CAFs and si‐ATG5/si‐Beclin1 CAFs treated under normoxic and hypoxic conditions for 24 h. (N) IF detection of co‐expression of SRGN and LC3B in the stromal tissue of WPOI 3 and WPOI 4 samples. ns, not significant; *p < .05; **p < .01; ***p < .001.

KEGG enrichment analysis revealed that the phagosome pathway in CAFs was significantly enriched in OSCC tissues with high WPOI (Figure 3A). Subsequently, WB and qRT‐PCR showed that hypoxic treatment expression of autophagy‐related genes was elevated in CAFs, while no such phenomenon was observed in OSCC tissues (Figure 3B,C; Figure S3A). This may be due to the persistent activation of the AKT/ERK/mTOR pathway in OSCC, which strongly inhibits the occurrence of autophagy.^27^ Moreover, TME revealed a higher number of autophagosomes in hypoxic CAFs, accompanied by an increase in mitochondrial numbers and swelling of the endoplasmic reticulum (Figure 3D,E). Addition of the autophagy inhibitor 3‐methyladenine (3‐MA) to CAFs resulted in a significant decrease in autophagy‐related genes after hypoxia treatment, as shown by qRT‐PCR and WB (Figure 3F; Figure S3B,C).^28^ IF indicated that LC3B expression was significantly increased in CAFs under hypoxic conditions (Figure S3D,E). These results suggest that hypoxia treatment increases autophagy in CAFs.

Further verification of the relationship between autophagy levels in CAFs and SRGN secretion. qPCR showed that SRGN expression was significantly increased in CAFs after hypoxic treatment, whereas no such phenomenon was observed in OSCC cells (Figure S3F). In addition, hypoxia significantly increased SRGN expression in CAFs, as shown by qRT‐PCR, WB, and ELISA, with 3‐MA treatment blocking this effect (Figure 3F,G; Figure S3C). IF analysis confirmed that hypoxia enhanced co‐expression of SRGN and LC3B in CAFs, which was reduced by 3‐MA (Figure 3H; Figure S3J). Given the potential off‐target effects of 3‐MA, we validated our findings using the specific Vps34 inhibitor SAR405.^29^ Hypoxic treatment increased ATG5, LC3B, and SRGN levels, but SAR405 specifically reversed the induction of LC3B and SRGN without affecting ATG5, pinpointing Vps34 activity as the critical driver (Figure S3H,I). Complementing this pharmacological approach, genetic knockdown of ATG5 or Beclin 1 independently confirmed the autophagy pathway's role, as silencing either gene severely compromised both SRGN expression and secretion under hypoxia (Figure 3I–M). In OSCC tissues with high WPOI, SRGN and LC3B were co‐expressed and elevated (Figure 3K; Figure S3K). Taken together, these results suggest a positive correlation between SRGN secretion and autophagy in CAFs.

### 3.4 Hypoxia‐induced SRGN secretion in CAFs via secretory autophagy

**FIGURE 4:**
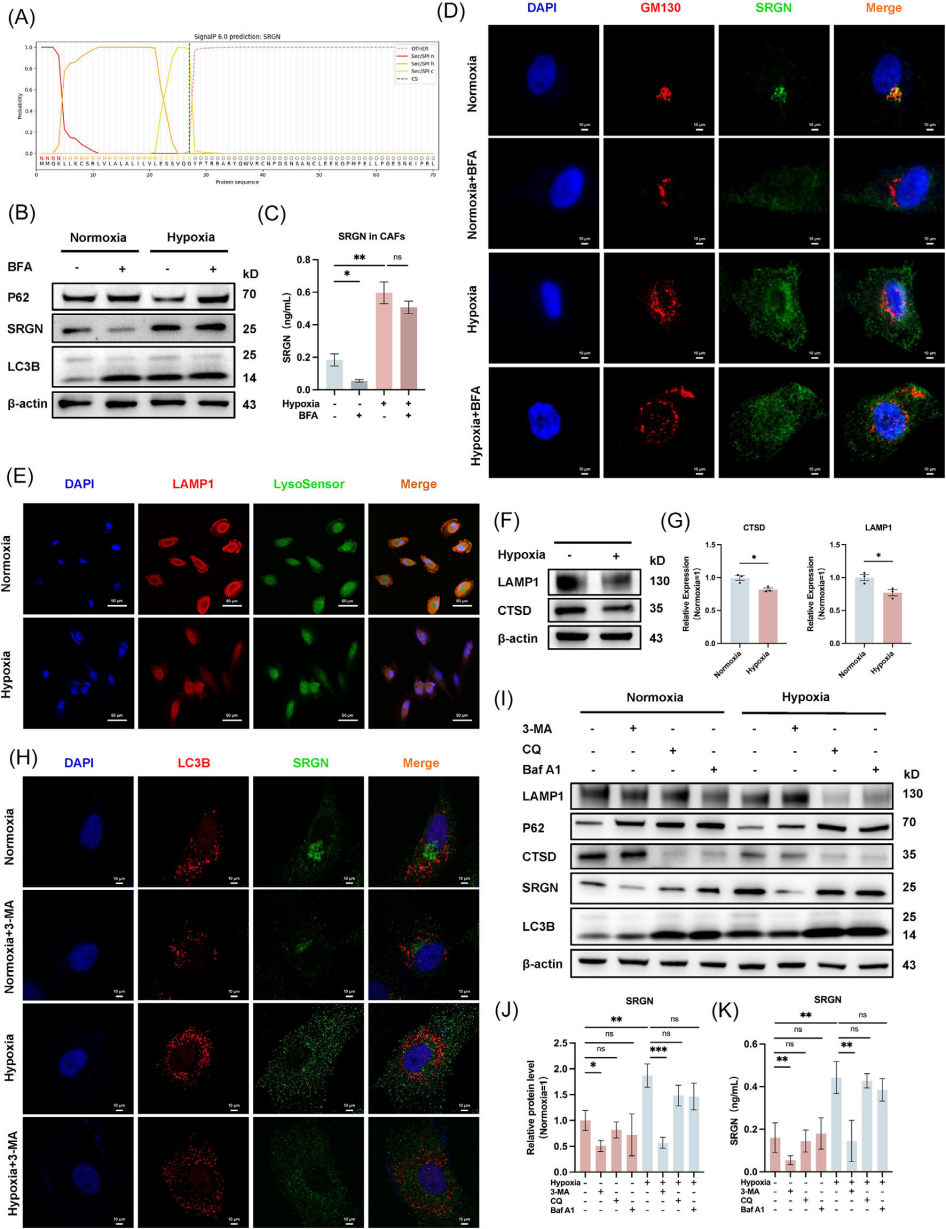
Under hypoxia, most SRGN is secreted extracellularly via the secretory autophagy pathway in CAFs. (A) Prediction of SRGN signal peptide structure using SignalP 6.0. (B) WB analysis of SRGN expression in normoxic and hypoxic CAFs with or without BFA (.2 µM, 24 h). (C) Detection of SRGN secretion levels in the supernatant of normoxic and hypoxic CAFs with or without BFA by ELISA. (D) IF showing co‐localization of SRGN and GM130 in normoxic and hypoxic CAFs with or without BFA. Scale bar = 10 µm. (E) IF observation of LAMP1 protein expression in normoxic and hypoxic CAFs and changes in LysoSensor intensity. Scale bar = 50 µm. (F, G) WB analysis of LAMP1 and CTSD protein expression levels in normoxic and hypoxic CAFs, along with quantitative analysis. (H) IF showing co‐localization of SRGN and LC3B in normoxic and hypoxic CAFs with or without 3‐MA. Scale bar = 10 µm. (I, J) WB analysis of LAMP1, P62, CTSD, SRGN, and LC3B protein expression levels in normoxic and hypoxic CAFs treated with BFA, CQ (60 µM), and Baf A1 (20 nM) for 24 h, along with quantitative analysis of SRGN expression. (K) Detection of SRGN secretion levels in the supernatant of normoxic and hypoxic CAFs treated with CQ and Baf A1 by ELISA. ns, not significant; *p < .05; **p < .01; ***p < .001.

Most eukaryotes employ N‐terminal signal peptides to direct proteins into the ER‐Golgi secretory pathway for conventional secretion.^30^, ^31^ Bioinformatics analysis using SignalP 6.0 on the eukaryotic SRGN sequence (NCBI database) confirmed the presence of this characteristic N‐terminal signal peptide (Figure 4A). To investigate SRGN's secretory pathway, we employed Brefeldin A (BFA), an inhibitor of ER‐to‐Golgi transport.^32^ Surprisingly, while BFA reduced SRGN expression under normoxia, hypoxia‐induced SRGN expression was unaffected (Figure 4B). Subsequently, further validation was obtained through ELISA experiments (Figure 4C). Furthermore, we performed IF labelling of GM130 and SRGN, which showed strong Golgi association of SRGN under normoxia, but hypoxic conditions led to SRGN overexpression with partial, BFA‐resistant Golgi co‐localization (Figure 4D).

Impaired lysosomal integrity is a key feature of secretory autophagy and critically regulates nonclassical protein secretion. ^33^ We incubated normoxic and hypoxic cells with the LysoTracker probe, and fluorescence intensity analysis showed that the acidic environment within the lysosomal lumen was disrupted after hypoxic treatment (Figure 4E; Figure S4A). WB results confirmed that hypoxia treatment significantly reduced both LAMP1 and CTSD levels (Figure 4F,G).

To distinguish between degradation and secretion autophagy, we treated CAFs with autophagy inhibitors: 3‐MA initiates phase inhibition; chloroquine (CQ) and bafilomycin A1(Baf‐A1) impair fusion with lysosomes and endosomal acidification (Figure S4B).^28^, ^34^, ^35^ CCK8 determined optimal drug concentrations (Figure S4C–E). IF confirmed stronger co‐localization of SRGN and LC3B after hypoxia (Figure 4H). WB showed that CQ and Baf‐A1 blocked autophagic flux and lysosomal damage under both conditions. Notably, 3‐MA suppressed SRGN expression in hypoxic CAFs, while CQ and Baf‐A1 had no effect (Figure 4I–J). Subsequently, Elisa produced the same result (Figure 4K). These results indicate that hypoxia‐induced SRGN secretion relies on autophagy initiation but not lysosomal degradation or the exosomal pathway.

### 3.5 SRGN‐mediated ECM remodelling and OSCC invasion via hypoxic autophagy in CAFs

**FIGURE 5:**
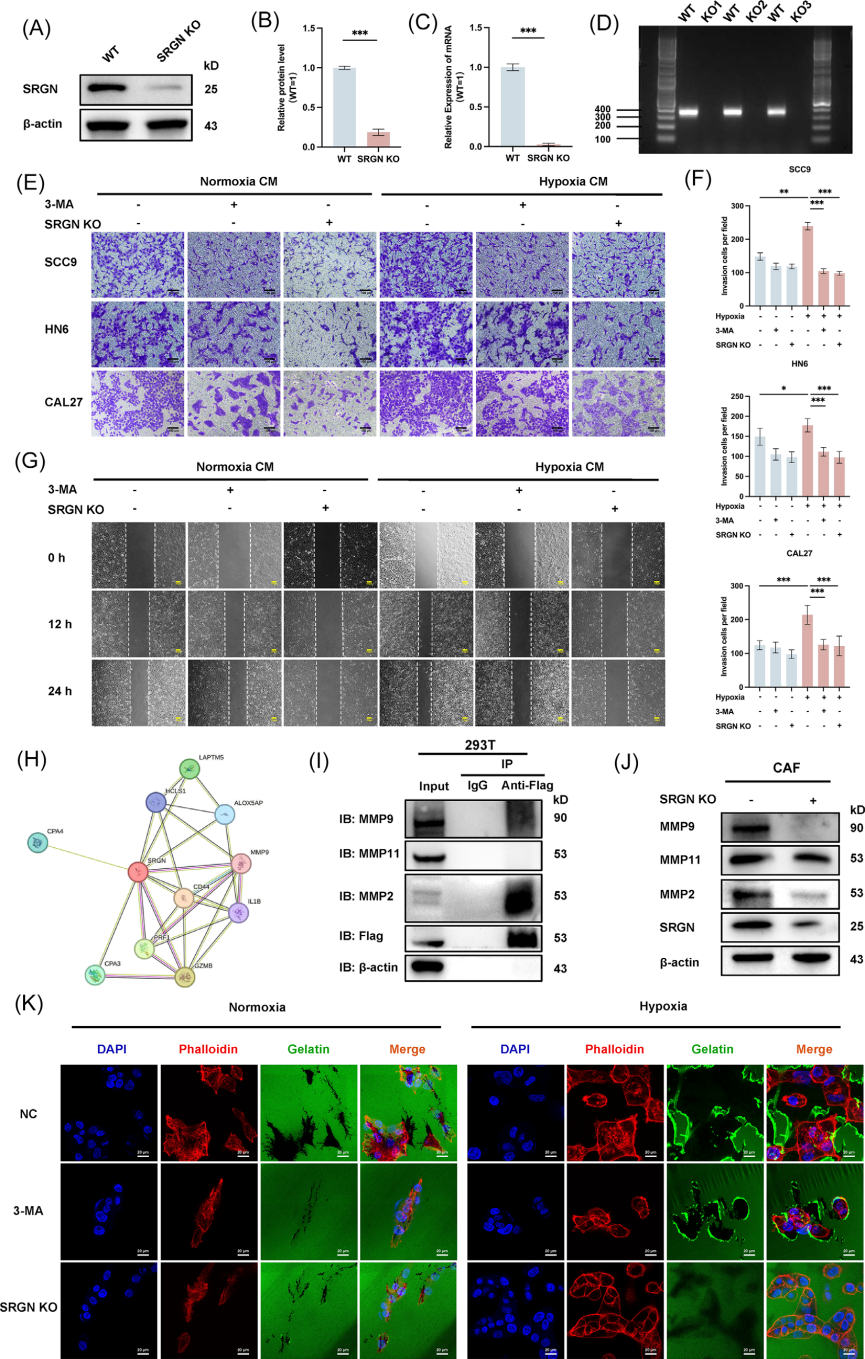
CAFs secrete SRGN via autophagy to promote OSCC cell invasion and migration by facilitating ECM remodelling through interaction with MMP2/9. (A, B) WB analysis of SRGN protein expression levels and quantification in WT CAFs and SRGN KO CAFs. (C) qPCR analysis of SRGN gene expression in WT CAFs and SRGN KO CAFs. (D) UV image of the agarose gel. (E) The supernatant from normoxic and hypoxic WT CAFs, WT CAFs + 3‐MA, and SRGN KO CAFs was collected and co‐cultured with OSCC cells. Invasion ability was assessed by transwell assays. (F) Invasion cell numbers were quantified using ImageJ software. (*p < .05; **p < .01; ***p < .001). (G) The supernatant from normoxic and hypoxic WT CAFs, WT CAFs + 3‐MA, and SRGN KO CAFs was collected and co‐cultured with SCC9 cells. Migration ability was assessed by scratch assays. (H) Prediction of SRGN‐binding proteins using the STRING database. (I) HEK293T cells were transfected with SRGN‐Flag and incubated for 48 h. Cell lysates were incubated with anti‐Flag beads, and immunoblotting (IB) was performed using anti‐Flag, anti‐MMP11, anti‐MMP9, and anti‐MMP2 antibodies. (J) WB analysis of changes in MMP9, MMP11, MMP2, and SRGN protein expression levels in WT CAFs and SRGN KO CAFs. (K) Gelatin degradation assays were performed to evaluate gelatin degradation after 24 h of co‐culture of CAL27 cells with the supernatants from normoxic and hypoxic WT CAFs, WT CAFs + 3‐MA, and SRGN KO CAFs. Scale bar = 20 µm.

To determine the effect of SRGN secreted by CAFs in OSCC progression, we established SRGN KO CAFs using CRISPR/Cas9‐mediated gene editing, with knockout efficiency validated by qRT‐PCR, WB and agarose gel electrophoresis (Figure 5A–D). CCK‐8 analysis revealed that hypoxia and 3‐MA did not significantly affect the proliferation of CAFs within 24 hours (Figure S5A,B). Transwell assays showed that hypoxia‐treated WT CAF CM markedly enhanced the invasive capacity of all three OSCC cell lines, as evidenced by increased cell penetration through the Matrigel‐coated membrane (Figure 5E,F). In contrast, CM from 3‐MA‐treated or SRGN KO CAFs significantly attenuated this pro‐invasive effect. Further assessed cell migration using scratch wound assays with SCC9 cells. Compared with 3‐MA‐treated and SRGN KO groups, hypoxia‐conditioned WT CAF CM accelerated wound closure, demonstrating enhanced migratory capacity (Figure 5G; Figure S5C). These findings collectively indicate that autophagy‐mediated SRGN secretion by hypoxic CAFs promotes both invasion and migration of OSCC cells.

Matrix metalloproteinases (MMPs) are essential components of the ECM, playing key roles in ECM remodelling.^26^, ^36^, ^37^ The STRING database was used to predict and validate the SRGN‐interacting proteins (Figure 5H). We found that among the SRGN‐interacting proteins, MMP9 was closely associated with ECM remodelling. Therefore, we overexpressed FLAG‐tagged SRGN (F‐SRGN) in 293T cells, and Co‐IP showed that SRGN interacted with both MMP2 and MMP9, but not with MMP11 (Figure 5I). Subsequently, further validation was performed in SRGN KO CAF by WB (Figure 5J). Further explore the impact of SRGN secreted by CAFs on the ECM degradation ability of OSCC cells. Notably, the gelatin matrix degradation increased significantly in CAL27 cells co‐cultured with the supernatant of WT CAFs from the hypoxia group, while autophagy inhibition or SRGN knockout resulted in a marked reduction in degradation (Figure 5K; Figure S5D). Cell–ECM adhesion is a critical step in distant metastasis of cancer^38^, ^39^ We found that the ECM adhesion capacity was significantly increased when co‐cultured with the supernatant of WT CAFs from the hypoxia group, but this effect was counteracted by the addition of 3‐MA or SRGN KO (Figure S5E). These results suggest that in the presence of SRGN, CAFs promoted the adhesion and degradation capacity of OSCC cells to ECM components.

### 3.6 CAFs promote tumour invasion and ECM degradation via secretory autophagy in vivo

**FIGURE 6:**
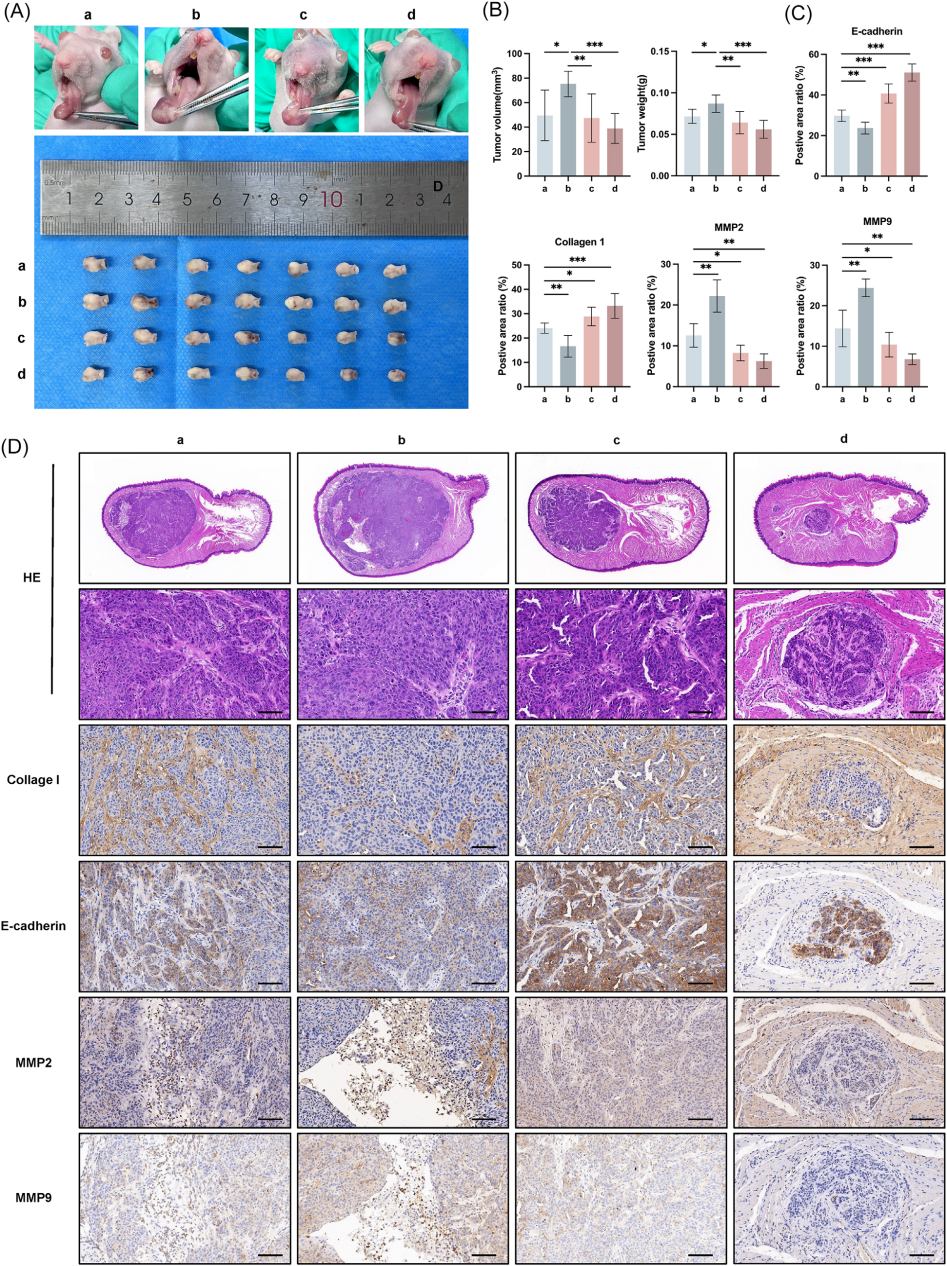
CAF‐derived SRGN promotes tumour invasion and ECM degradation via autophagy secretion. (A) In vivo xenograft models were established in nude mice and divided into four groups: (a) CAL27, (b) CAL27 + WT CAFs, (c) CAL27 + WT CAFs (3‐MA), and (d) CAL27 + SRGN KO CAFs. (B) Tumour volume and tumour weight were monitored (*n* = 7). (C, D) H&E staining and IHC analysis of COL1, E‐cadherin, MMP2 and MMP9 were performed in orthotopic xenograft tumour tissues. The expression levels of COL1, E‐cadherin, MMP2 and MMP9 were quantitatively analyzed using Fiji software. Scale bar = 100 µm. (*p < .05; **p < .01; ***p < .001).

To assess CAFs' impact on tumour invasion in vivo, we created orthotopic xenografts using nude mice randomized into four groups: (a) CAL27; (b) CAL27 + WT CAFs; (c) CAL27 + WT CAFs treated with 3‐MA; (d) CAL27 + SRGN‐KO CAFs. After two weeks, tumour analysis revealed that WT CAFs significantly promoted CAL27 tumour growth compared with CAL27 alone, but were markedly suppressed by either 3‐MA or SRGN KO (Figure 6A,B). Histological analysis showed Group (b) displayed nuclear atypia, dedifferentiation and central necrosis, indicating higher proliferative, invasive potential and malignant grade. The invasive potential of tumours in each group was assessed through IHC staining. The results showed that in group (b), E‐cadherin expression was significantly downregulated, Collagen I showed obvious degradation, and extensive infiltration of MMP2/MMP9 was observed in the necrotic areas, which coincided with the region of active ECM remodelling, suggesting increased invasiveness (Figure 6C,D). However, this enhanced invasiveness could be reversed by 3‐MA treatment or SRGN knockout. Notably, although in vitro WB demonstrated that the autophagic inhibition by 3‐MA diminished within 24–48 h after drug withdrawal (Figure S6A), its tumour‐suppressive effect in vivo persisted. Through ELISA analysis normalized by cell counting, we found that CAFs maintained significantly suppressed SRGN secretion capacity even after autophagic flux had recovered (Figure S6B). Collectively, these results demonstrate that CAFs promote OSCC growth and invasion in vivo via autophagy‐mediated SRGN secretion, and that short‐term intervention targeting this pathway can generate sustained tumour‐suppressive outcomes.

## 4 DISCUSSION

Current understanding of the pathobiological mechanisms driving distinct WPOI patterns and their association with poor prognosis in OSCC remains limited. Through scRNA‐seq analysis, we discovered that CAFs were significantly enriched in highly infiltrated OSCC samples. Besides, the SRGN+ myCAF terminal activation state was identified as a differentiation endpoint in myCAFs, which suggests that SRGN in CAFs may be more abundant in advanced tumours or metastatic foci, and could be correlated with patient prognosis. As a secreted proteoglycan, SRGN plays a pivotal role in invasive metastasis and immune escape in several cancers, including non‐small‐cell lung cancer (NSCLC), breast cancer, and OSCC.^13^, ^40^, ^41^

Our study demonstrates that hypoxic conditions significantly enhance autophagic activity in CAFs, with both expression and secretion levels of SRGN showing positive correlation with autophagic flux. Notably, pharmacological inhibition of autophagy using 3‐MA/SAR405 or genetic knockdown of ATG5/Beclin1 substantially reduced SRGN levels in both intracellular and extracellular compartments, indicating autophagy plays a central role in SRGN regulation. Mechanistically, autophagy appears to regulate SRGN through two distinct pathways: first, by sustaining an autocrine positive feedback loop where extracellular SRGN activates NF‐κB/MAPK signalling through its receptor, thereby promoting its own transcription^42^, ^43^, ^44^; second, complete autophagy blockade leads to accumulation of damaged organelles and misfolded proteins, triggering ER stress and subsequent PERK–eIF2α pathway activation that globally suppresses protein synthesis, including SRGN expression.^45^, ^46^ These findings collectively reveal a dual mechanism through which autophagy precisely controls SRGN expression and function via both secretory regulation and cellular homeostasis maintenance, providing novel molecular insights into autophagy‐mediated secretome homeostasis in the tumour microenvironment.

Existing studies have reported that CAFs can promote HNSCC progression through secretory autophagy‐mediated release of IL‐6 and IL‐8.^47^ Here, we identify a novel key effector molecule secreted by CAFs through this pathway. As a secretory protein with an N‐terminal signal peptide, SRGN typically enters the extracellular matrix through the conventional secretion pathway under normoxic conditions. However, under hypoxic conditions, its secretion shifts to a non‐classical pathway that does not rely on Golgi transport. Various proteins released via secretory autophagy, such as diazepam binding inhibitor/Acyl‐CoA‐binding protein/α‐centractin (DBI/ACBP/AcbA), CFTRΔF508, IL1B, and Annexin A2 (ANXA2), adopt similar mechanisms.^48^, ^49^, ^50^, ^51^, ^52^ Secretory autophagy shares the autophagosome formation stage with degradative autophagy, but the former directly releases its contents by fusion of the autophagosome with the plasma membrane.^53^ In this study, we observed significant lysosomal damage in hypoxic CAFs, which is consistent with reports that HIF‐1α downregulates ATP6V1A to impair lysosomal function in HNSCC.^54^ Importantly, CQ/Baf A1 interference with lysosomal function did not affect SRGN secretion, while inhibition of autophagosome formation with 3‐MA significantly blocked SRGN expression. These findings collectively demonstrate that hypoxic CAFs specifically mediate SRGN extracellular release through secretory autophagy. Notably, in normoxic CAFs, CQ and BAF A1 treatments significantly reduced CTSD expression, a phenomenon consistent with feedback regulation triggered by impaired lysosomal function. Continuous inhibition of the autophagy‐lysosome pathway leads to the accumulation of undegraded substrates within the lysosome, which may trigger an adaptive response through the mTOR‐TFEB signalling axis, thereby downregulating the expression of lysosomal hydrolases like CTSD to restore intracellular homeostasis.^55^, ^56^

In oesophageal squamous cell carcinoma (ESCC) and glioblastoma (GBM), SRGN can indirectly upregulate the expression of MMP2/MMP9 by activating the ERK pathway, TGF‐β signalling, or the CXCLs/CXCR2 axis, thereby enhancing tumour invasion and metastasis.^57^, ^58^ Our study further confirms that SRGN can directly interact with MMP2/MMP9 to promote ECM remodelling. We propose that SRGN, as a proteoglycan carrying abundant chondroitin sulfate/heparin (CS‐E, HP) glycosaminoglycan chains, can form stable complexes with MMP2/MMP9 in the ECM.^13^ On one hand, this protects them from endogenous inhibitors and delays their degradation, thereby extending enzyme activity duration.^59^ On the other hand, SRGN acts as a scaffold protein to concentrate MMPs at specific action sites, enhancing the local degradation efficiency of ECM.^60^ It is noteworthy that SRGN knockout leads to a significant decrease in MMP2/9 expression, suggesting that its role goes beyond simple binding and constitutes a complete signal amplification loop. Specifically, the SRGN‐MMPs complex degrades ECM, releasing growth factors (such as TGF‐β) and exposing cryptic epitopes, which in turn activate downstream TGF‐β/SMAD and ERK signalling pathways via integrin receptors.^61^, ^62^ The SRGN‐MMP axis establishes a self‐reinforcing cycle: SRGN activates MMPs to remodel the ECM, which in turn triggers signalling pathways that enhance MMPs’ transcription. Disruption of this circuit simultaneously reduces MMPs gene expression and protein production, confirming its central role in controlling OSCC invasion

In our xenograft model, co‐transplantation of CAFs and CAL27 cells induces downregulation of E‐cadherin expression and degradation of COL1, suggesting a decrease in tumour differentiation and enhanced invasiveness.^63^, ^64^ This pro‐tumour effect can be significantly reversed by 3‐MA pretreatment or SRGN KO. It is worth noting that although 3‐MA's inhibitory effect on autophagy is temporary in vitro, it exerts a sustained anti‐tumour effect in vivo. Previous studies have reported that short‐term strong stimuli can induce epigenetic remodelling of stromal cells, including changes in DNA methylation and histone modifications, leading to a persistent alteration in their functional state, a phenomenon known as “stromal cell functional reprogramming”.^65–68^ We hypothesize that the 24 h 3‐MA pretreatment may disrupt the positive feedback loop between CAFs and tumour cells by acutely interrupting SRGN secretion, leading to a sustained transition of CAFs to a low‐activity state. This could explain why, despite the recovery of autophagic function in vitro, CAFs in vivo are still unable to fully restore their pro‐tumour capacity.

The findings of this study provide important directions for further exploration. First, the precise molecular mechanism through which hypoxia regulates SRGN via secretory autophagy remains to be fully elucidated, particularly the specific recognition of SRGN by autophagosomes and the subsequent exocytosis process, which is still unclear. Second, the immune‐deficient mouse model used in the current study has limitations, as it cannot accurately assess the regulatory function of SRGN in the tumour immune microenvironment. Future research should establish immune‐competent animal models (such as C57BL/6 mice) to clarify the specific role of SRGN derived from CAFs in regulating anti‐tumour immune responses. On the translational medicine front, conducting preclinical intervention studies targeting the SRGN pathway holds significant value. We suggest targeting the SRGN pathway in immune‐competent animal models to evaluate its anti‐tumour efficacy. This could include the use of SRGN neutralizing antibodies or clinically relevant autophagy inhibitors (such as 3‐MA/SAR405) to determine whether blocking this pathway can inhibit tumour growth, metastasis, and possibly reverse immune suppression, thereby providing strong evidence for subsequent clinical trials.

## 5 CONCLUSION

**FIGURE 7:**
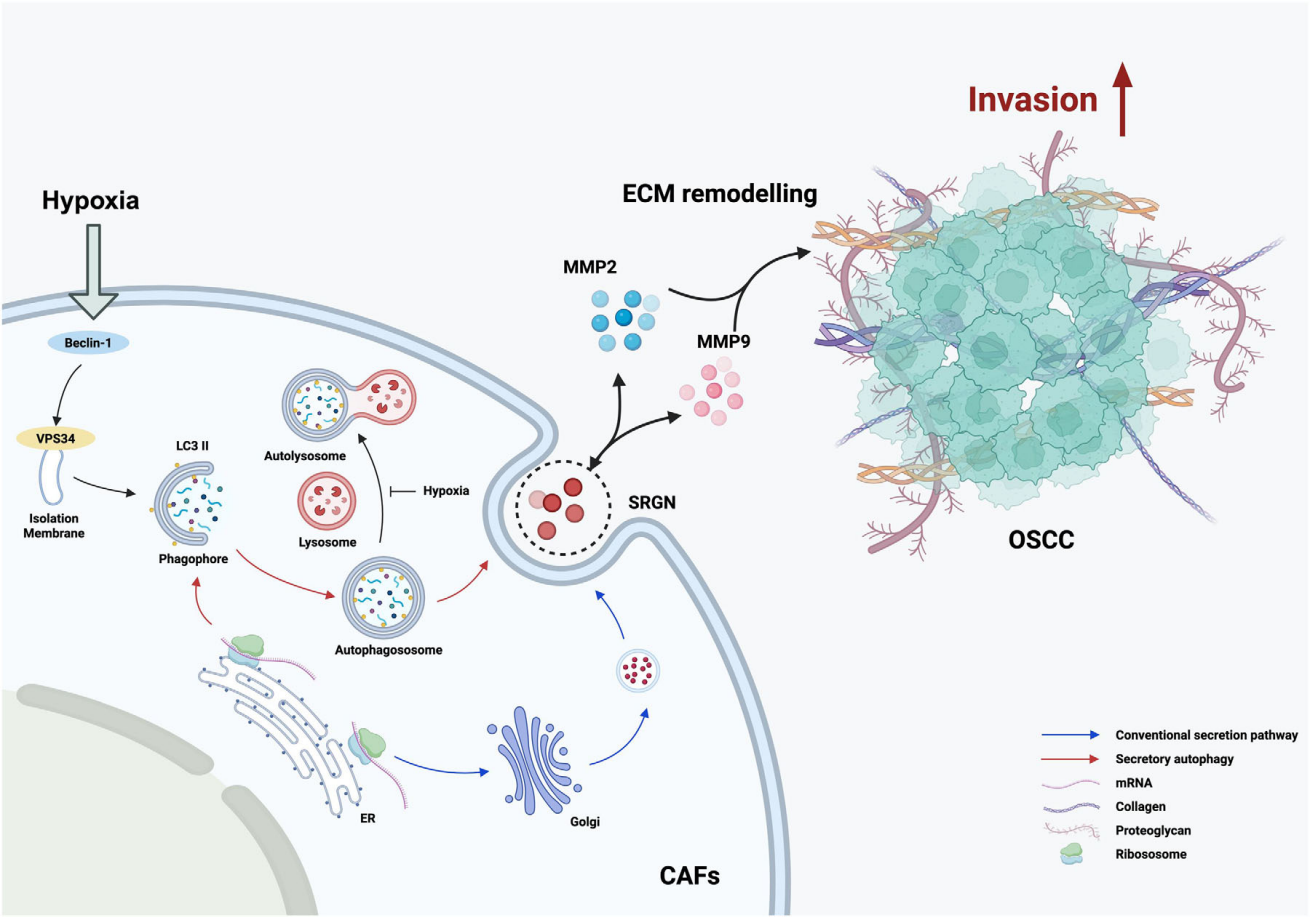
Mechanism diagram of hypoxic CAFs‐derived SRGN secretion and tumour progression promotion. Under normal conditions, SRGN is translocated into the ER and subsequently transported via the Golgi apparatus for secretion into the extracellular space. Under hypoxic conditions, elevated autophagy levels in CAFs facilitate the release of SRGN into the ECM through secretory autophagy‐mediated plasma membrane fusion. Within the ECM, SRGN interacts with MMP2 and MMP9, enhancing ECM remodelling and ultimately promoting the invasive capacity of OSCC cells.

In summary, our study reveals that SRGN, a key cytokine secreted by CAFs in hypoxic OSCC microenvironments, exhibits autophagy‐dependent secretion. Specifically, under hypoxic conditions, SRGN was released into the ECM via the secretory autophagy pathway. Mechanistically, SRGN promotes ECM remodelling via direct interactions with MMP2/MMP9, thereby enhancing OSCC cell invasion and migration (Figure 7). In WPOI 4–5 type OSCC, developing effective strategies to knock down SRGN may help overcome tumour progression induced by SRGN secretion.

## AUTHOR CONTRIBUTIONS

Yan Zhang: visualization, validation, software, methodology, investigation, formal analysis, data curation, conceptualization and writing—original draft. Cheng Tao: visualization, validation, software, methodology, investigation, formal analysis, and writing—original draft. Xiteng Yin: methodology, data curation and writing—review & editing. Zhi Wang: software, methodology, data curation and writing—review & editing. Yuyang Zhang: formal analysis and writing—review & editing. Jiale Yu: writing—review & editing. Yufeng Wang: resources, project administration, funding acquisition and writing—review & editing. Wei Han: resources, project administration, funding acquisition and writing—review & editing.

## CONFLICT OF INTEREST STATEMENT

The authors declare no conflict of interest.

## ETHICS STATEMENT

The clinical study has been approved by the Nanjing Stomatological Hospital Ethics Committee (NJSH‐2025NL‐088). The animal experiments have received preapproval from the Institutional Animal Care and Use Committee (IACUC) of the Medical School of Nanjing University (IACUC—D2304022).

## Supporting information



Supporting information

Supporting information

## Data Availability

The data supporting the findings of this study are available from the corresponding author upon reasonable request.
