# Supplementary material for: Hypoxia‐induced secretory autophagy in cancer‐associated fibroblasts promotes ECM remodelling through serglycin secretion in oral squamous cell carcinoma
Source: Clin Transl Med. 2025 Dec 18;15(12):e70556. doi: 10.1002/ctm2.70556 (PMC12712735; doi:10.1002/ctm2.70556)
Supplement: Supplementary file 1 — Supporting information [file CTM2-15-e70556-s002.docx]

**
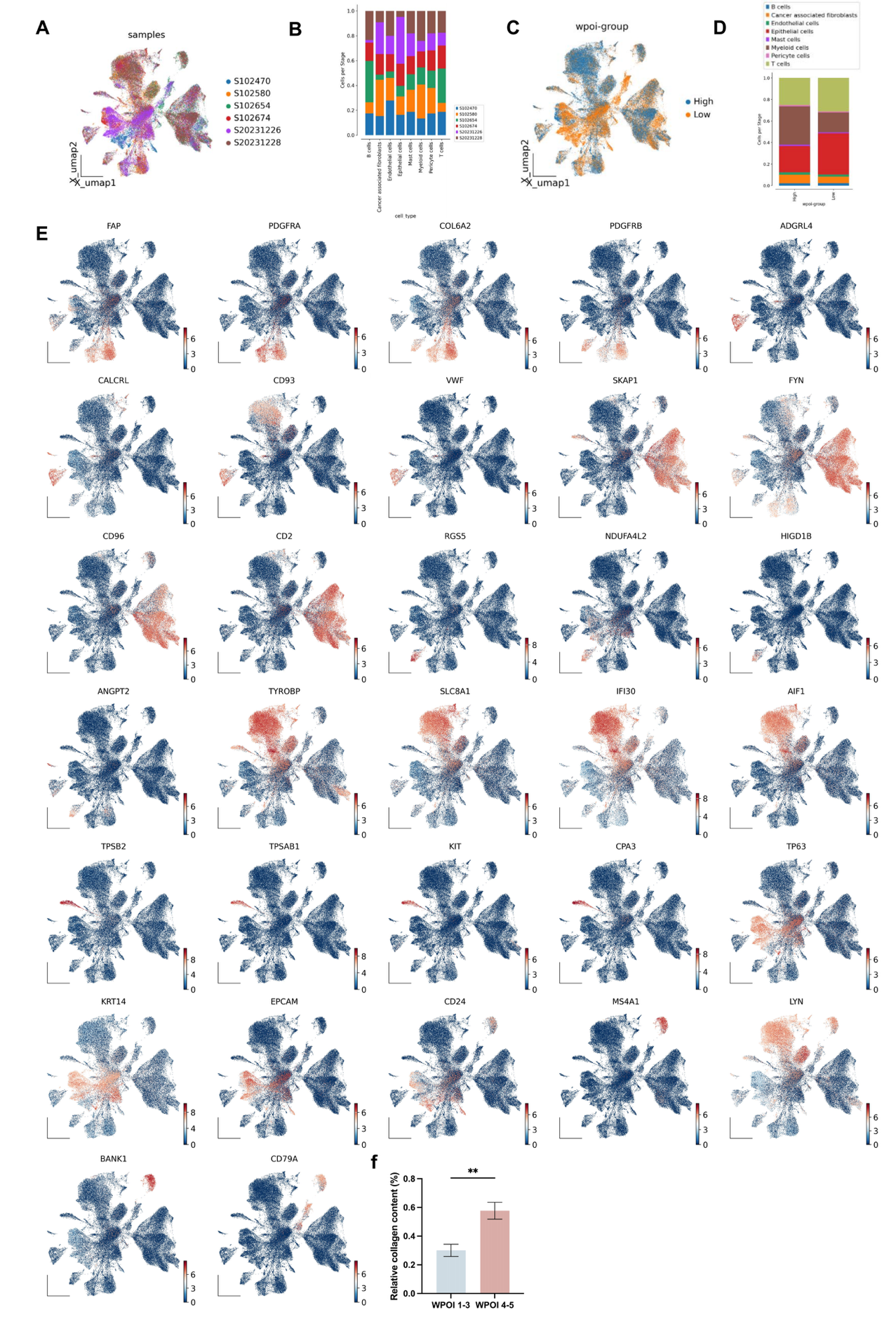
**

**Figure S1.** Batch effect correction and marker gene umap. (A) UMAP visualization of integrated samples after batch correction based on the Harmony method, colored by sample origin (n=6). The cellular composition of the samples is shown as a stacked bar graph showing the relative abundance of cell populations. (B) Cellular composition of the samples is shown as a stacked bar graph. UMAP projections are stratified by WPOI group (high vs. low). (C) Proportion of cell populations in each WPOI category is indicated (high: n=3; low: n=3). (D) Expression patterns of marker genes in 8 cell populations with gradient colors indicating normalized expression levels, examples of typical CAF markers COL6A2, FAP, PDGFRA, PDGFRB in CAFs. (E) Cell population markers and their umap distribution. (F) ImageJ software statistics of collagen content in Masson staining. (**P < 0.01).


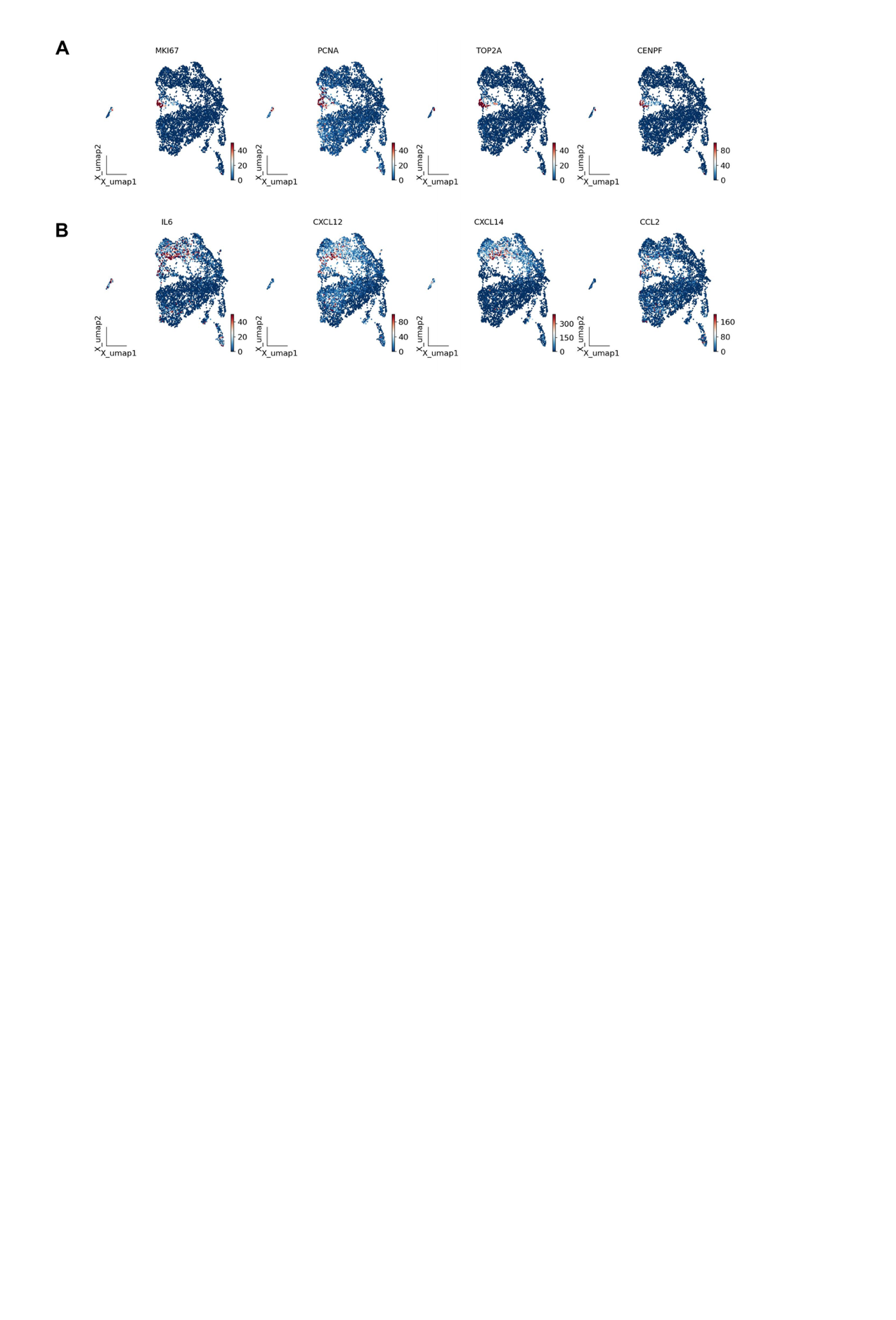


**Figure S2. Other subtypes of CAFs besides myCAFs. (A) proCAFs are in an active cell cycle, characterized by high expression of core genes directly related to cell proliferation, such as MKI67, PCNA, TOP2A, and CENPF. (B) iCAFs are characterized by high expression of IL6, CXCL12, CXCL14, CCL2, and other inflammation-related markers.**


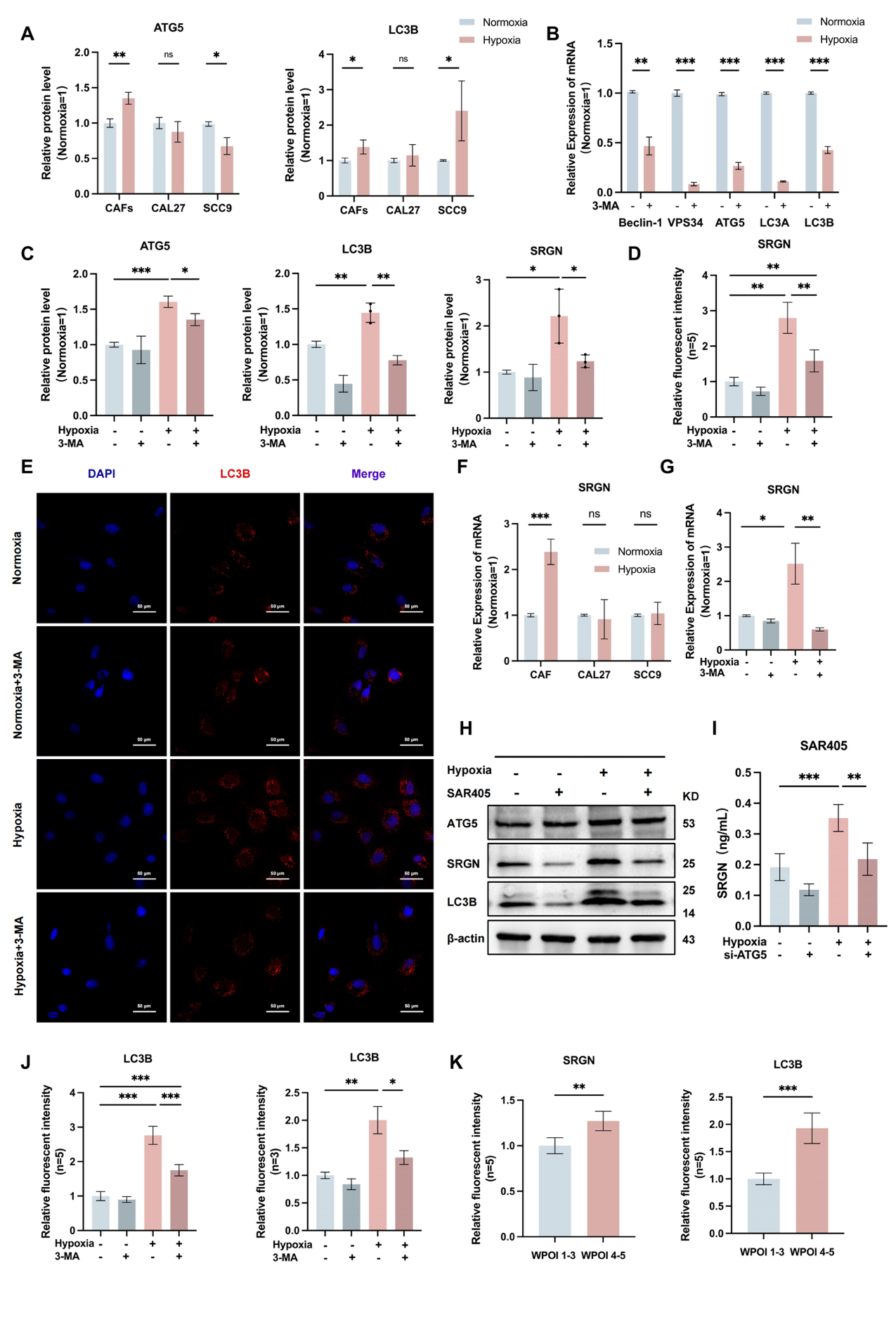


**Figure S3.** Hypoxia promotes increased autophagy levels in CAFs. (A) WB quantitative analysis of ATG5 and LC3B expression in CAF, CAL27, and SCC9 cells under normoxic and hypoxic conditions. (B) After adding 3-MA (5 mM, 24h) to the hypoxic group, autophagy markers in normoxic and hypoxic CAFs were assessed by qPCR. (C) WB quantitative analysis of ATG5, LC3B and SRGN expression in CAFs under normoxic and hypoxic conditions after 3-MA treatment, along with their quantitative analysis. (D-E) IF detection of LC3B expression in CAFs under normoxic and hypoxic conditions and after 5 mM 3-MA treatment, along with statistical analysis of relative fluorescence intensity. Scale bar = 50μm. (F) qPCR shows SRGN expression in CAFs, CAL27, and SCC9 under normoxic and hypoxic conditions. (G) qPCR shows SRGN expression in normoxic and hypoxic CAFs with or without 3-MA. (H) WB reveals changes in ATG5, LC3B and SRGN expression in CAFs under normoxic and hypoxic conditions after SAR405 treatment. (I) ELISA detection of SRGN secretion in the supernatant of normoxic and hypoxic CAFs, with or without SAR405 treatment. (J) The fluorescence intensities of LC3B and SRGN were quantified using Fiji software. (K) The fluorescence quantification of LC3B and SRGN in OSCC tissues with high and low WPOI grades. (ns, not significant; *P < 0.05; **P < 0.01; ***P < 0.001).


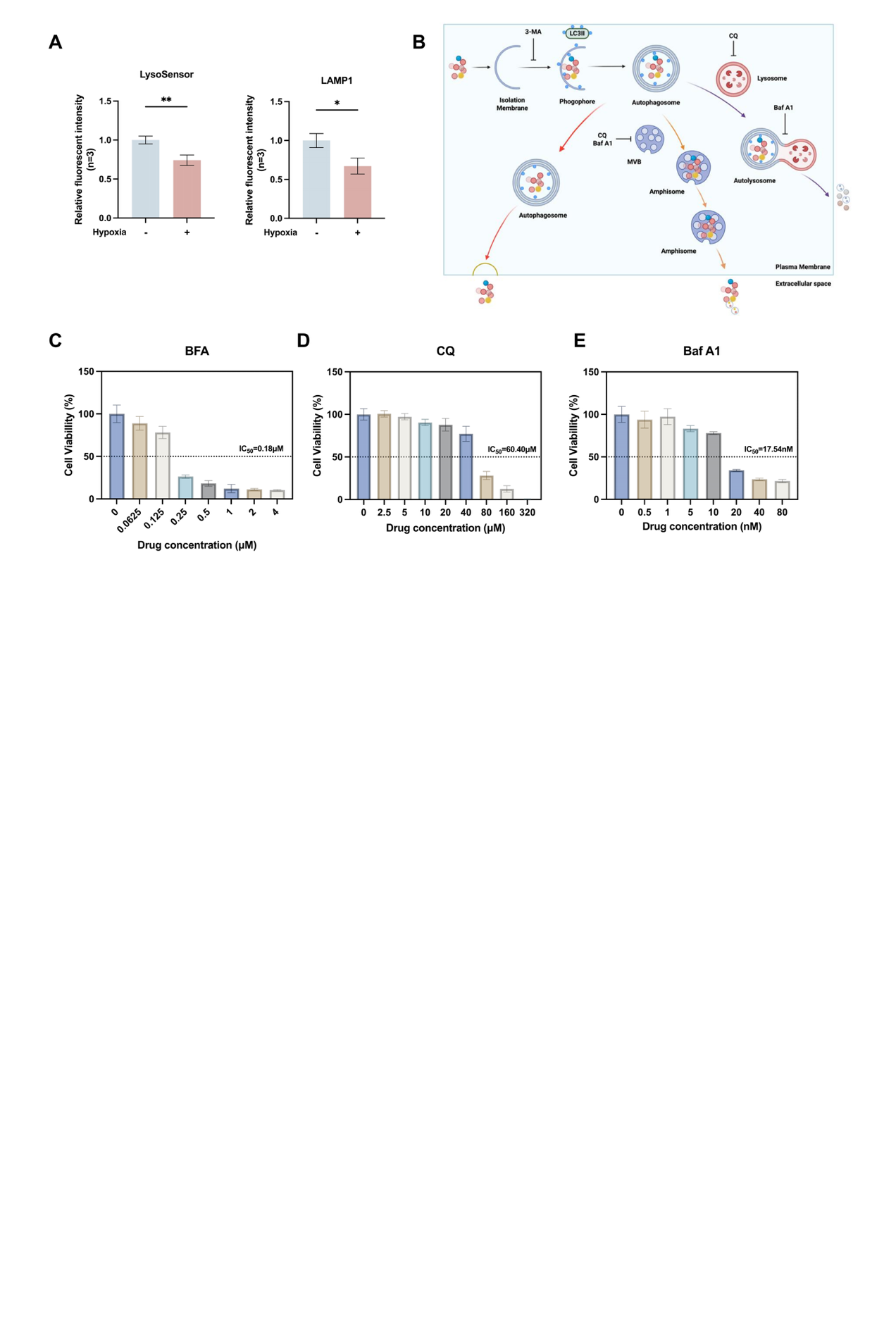


**Figure S4.** Hypoxic CAFs release SRGN through secretory autophagy. (A) Relative fluorescence intensity quantification of LysoSensor and LAMP1. (B) Schematic diagram of secretory autophagy mechanisms. The purple arrow represents degradative autophagy: autophagosomes fuse with lysosomes to form autolysosomes, where substrates are degraded and the products are released into the extracellular space. The yellow arrow represents the endosome-exosome pathway: exosomes are vesicles released through fusion of multivesicular bodies (MVB) with the plasma membrane. The red arrow represents secretory autophagy: autophagosomes do not fuse with lysosomes but directly fuse with the plasma membrane to release substrates into the extracellular space. (C-E) Optimal drug concentrations of BFA, CQ, and Baf A1 in CAFs were measured by CCK8. (*P < 0.05).


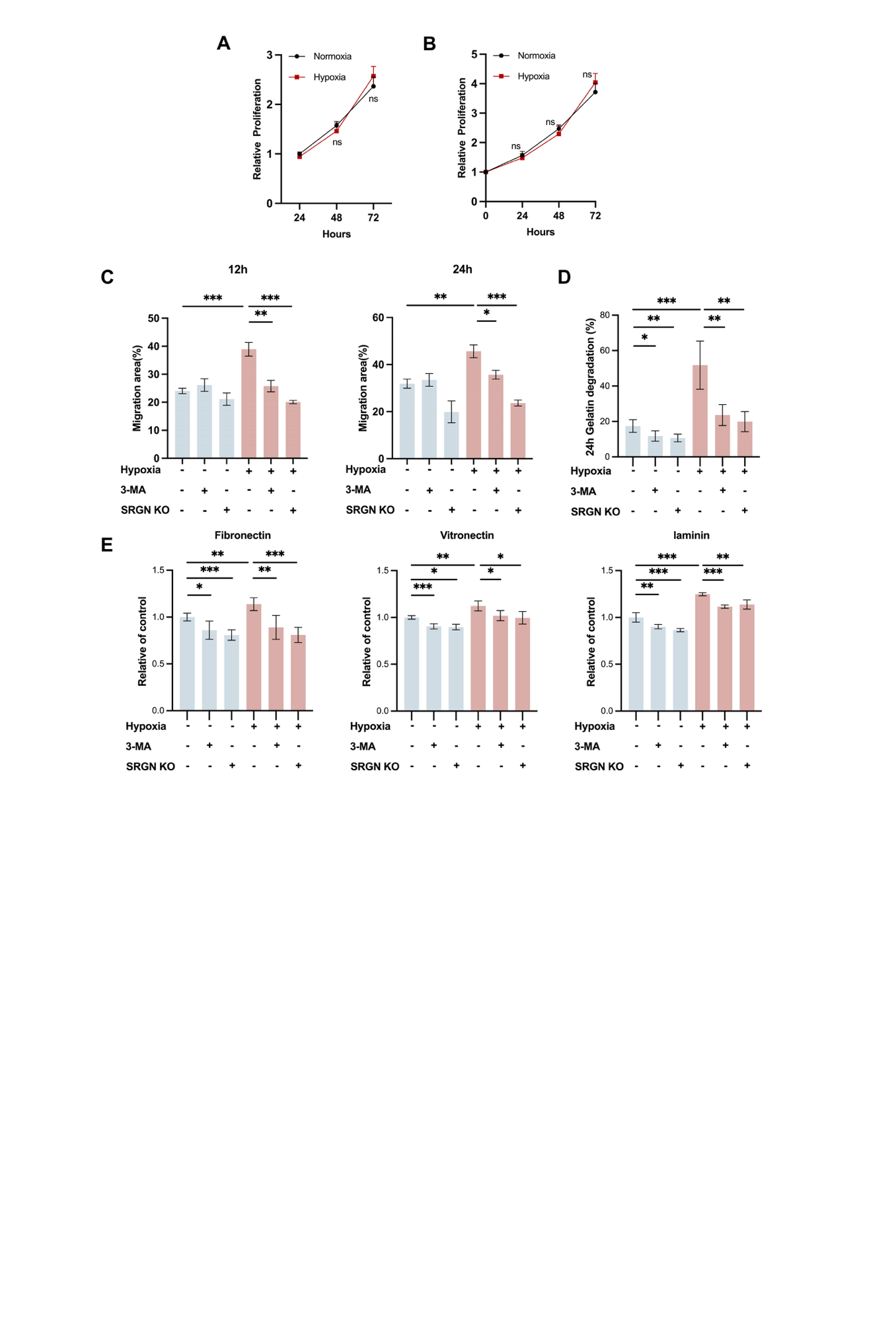


**Figure S5.** CAFs secrete SRGN via autophagy to promote OSCC cell invasion and migration by facilitating ECM remodelling. (A) CCK8 assay measured the cell viability of CAFs after hypoxia treatment. (B) CCK8 assay determined the cell viability of CAFs after the addition of 5 mM of 3-MA in normoxia and hypoxia. (C) The changes in wound width were measured using Image J software. (D) Gelatin degradation areas were quantified using Image J software (n = 5). (E) After 72 hours of adhesion in ECM-coated 12-well plates, the adhesion numbers to fibronectin, laminin, and vitronectin were measured using the CCK-8 assay. (ns, not significant; *P < 0.05; **P < 0.01; ***P < 0.001).


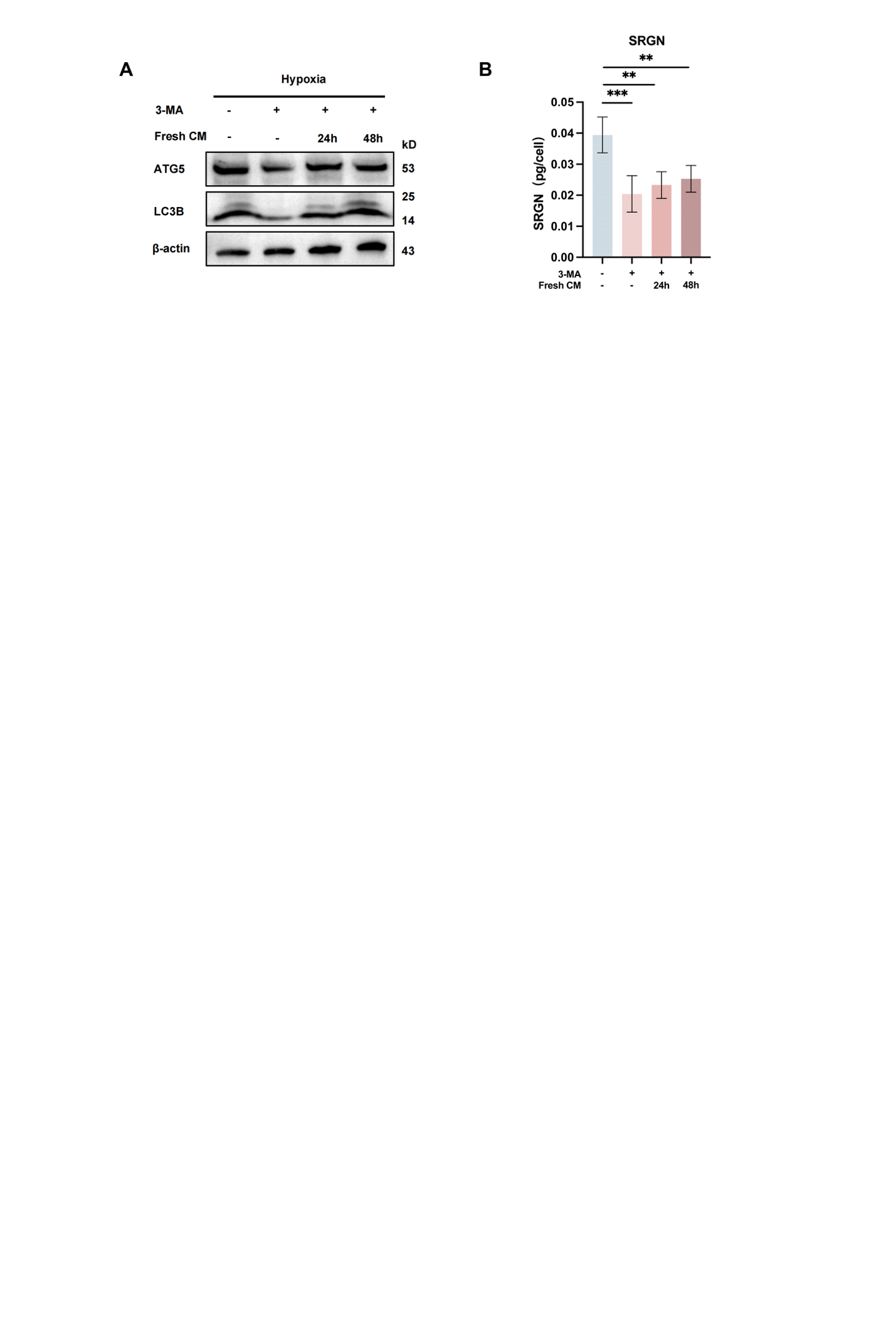


**Figure S6.** Effective duration of 3-MA in inhibiting CAFs. (A) CAFs were cultured under hypoxia, divided into control and 3-MA groups. After 24-hour treatment, 3-MA was removed. Fresh medium was replaced, and cells were cultured for an additional 24 or 48 hours. ATG5 and LC3B expression were then measured by WB. (B) Using the same culture and treatment groups as in (A), cells were counted after an additional 24 or 48 hours. The SRGN concentration in the supernatant was divided by the total cell count, and SRGN levels in the supernatant were measured by ELISA to calculate the SRGN secretion per cell.
