# Supplementary figures and images for: Hypoxia‐induced secretory autophagy in cancer‐associated fibroblasts promotes ECM remodelling through serglycin secretion in oral squamous cell carcinoma
Source: Clin Transl Med. 2025 Dec 18;15(12):e70556. doi: 10.1002/ctm2.70556 (PMC12712735; doi:10.1002/ctm2.70556)

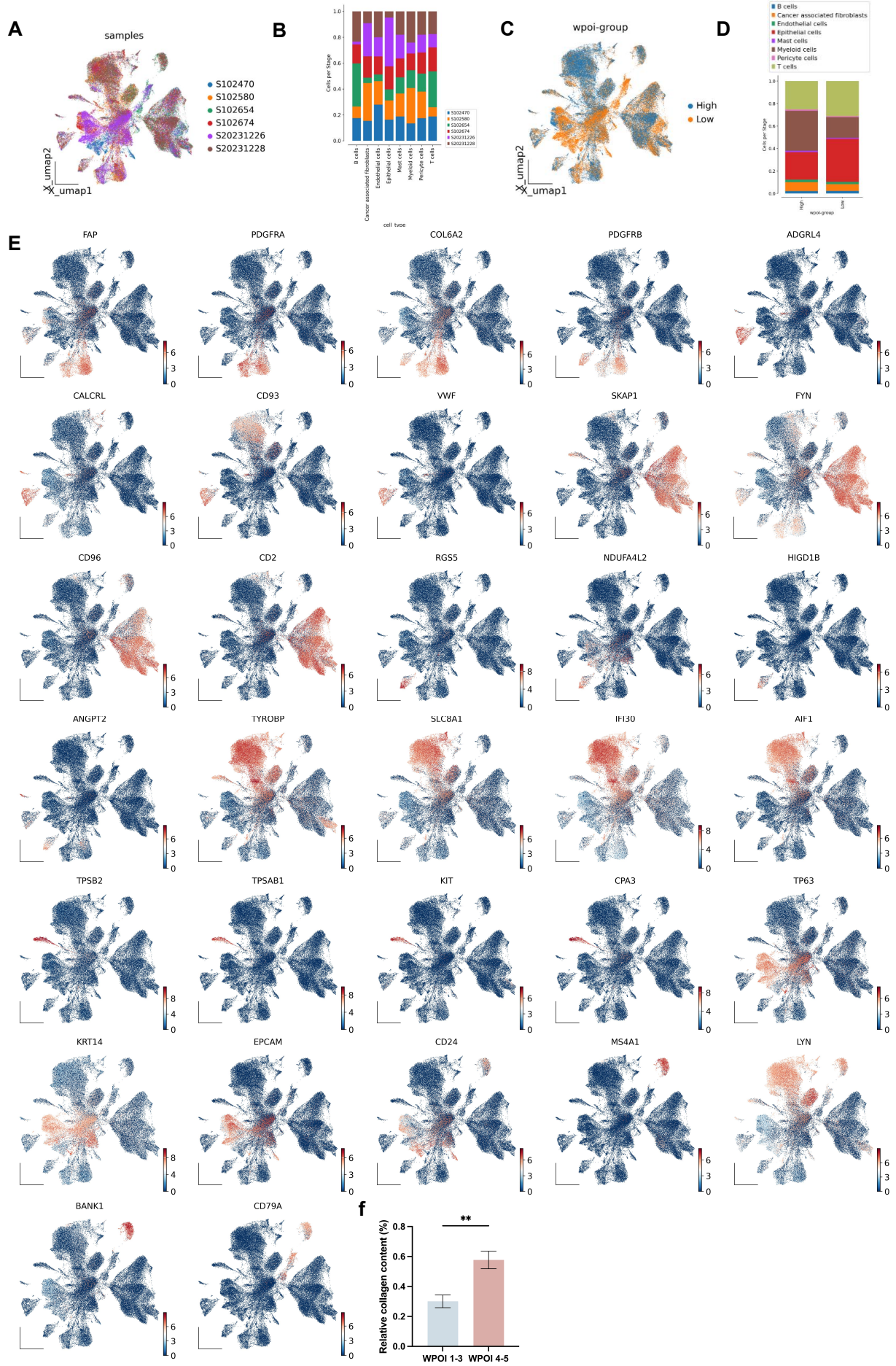

**A**

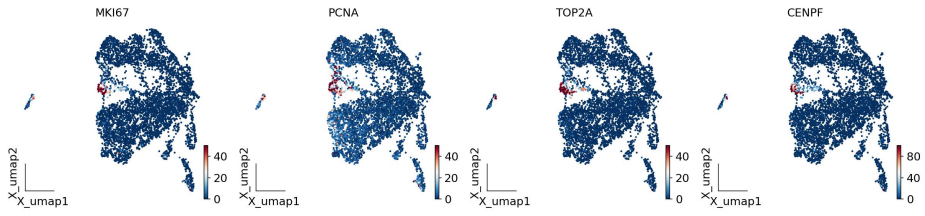

**B**

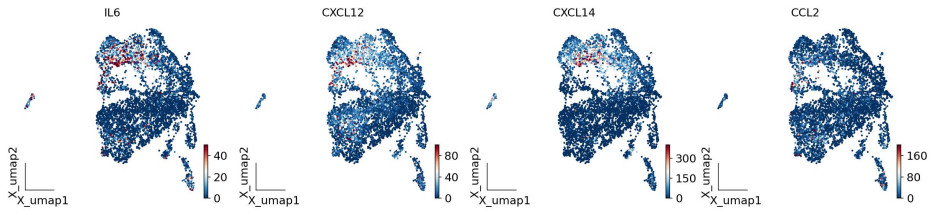

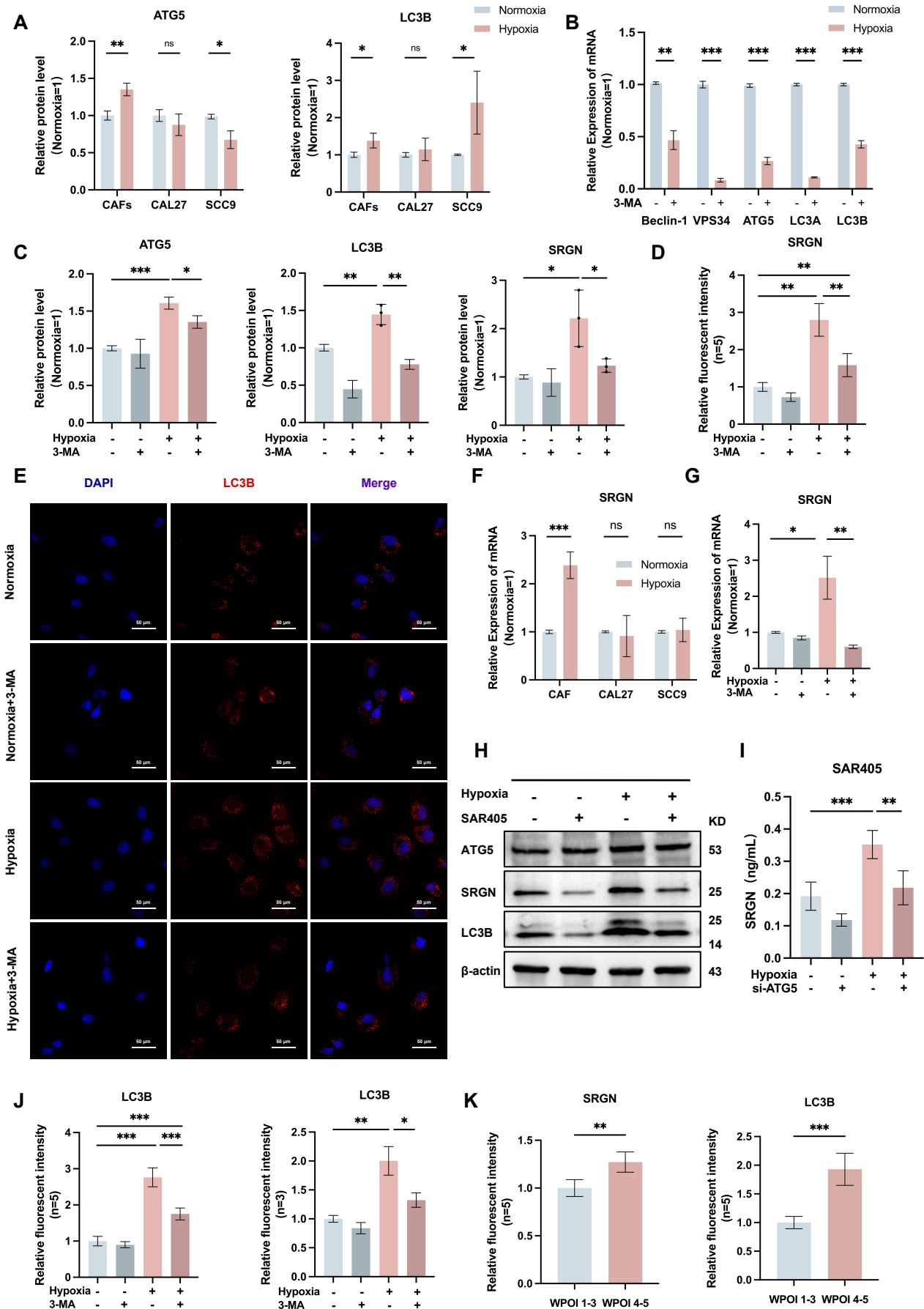

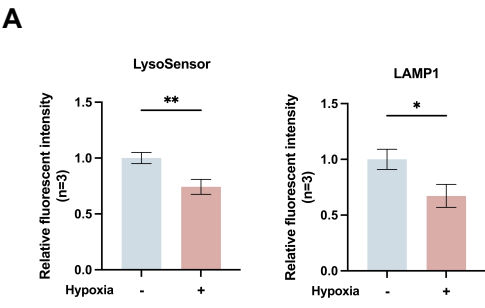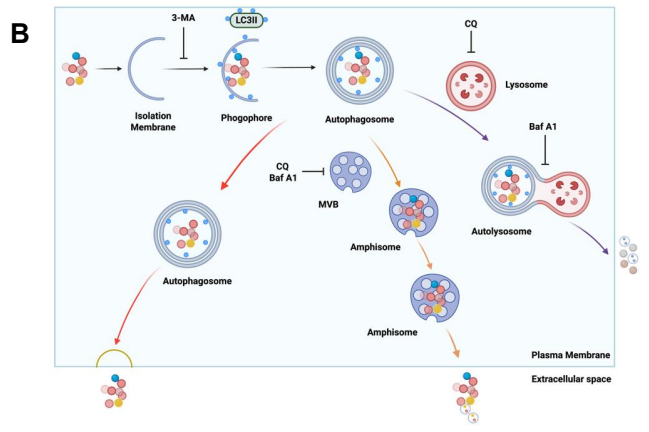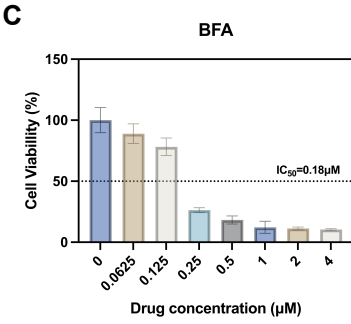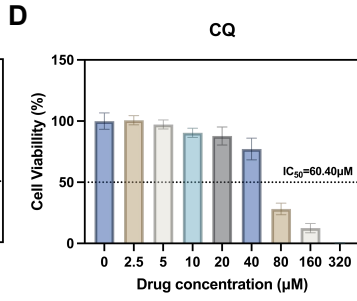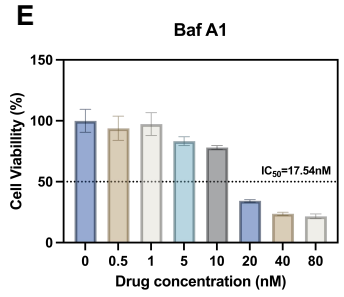

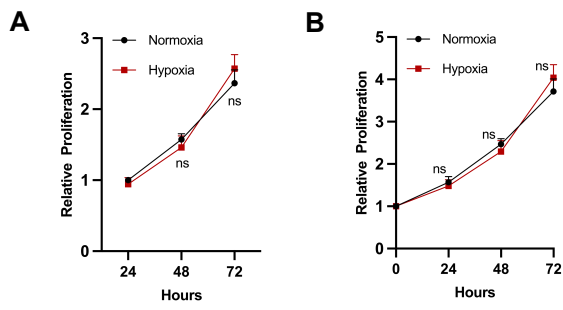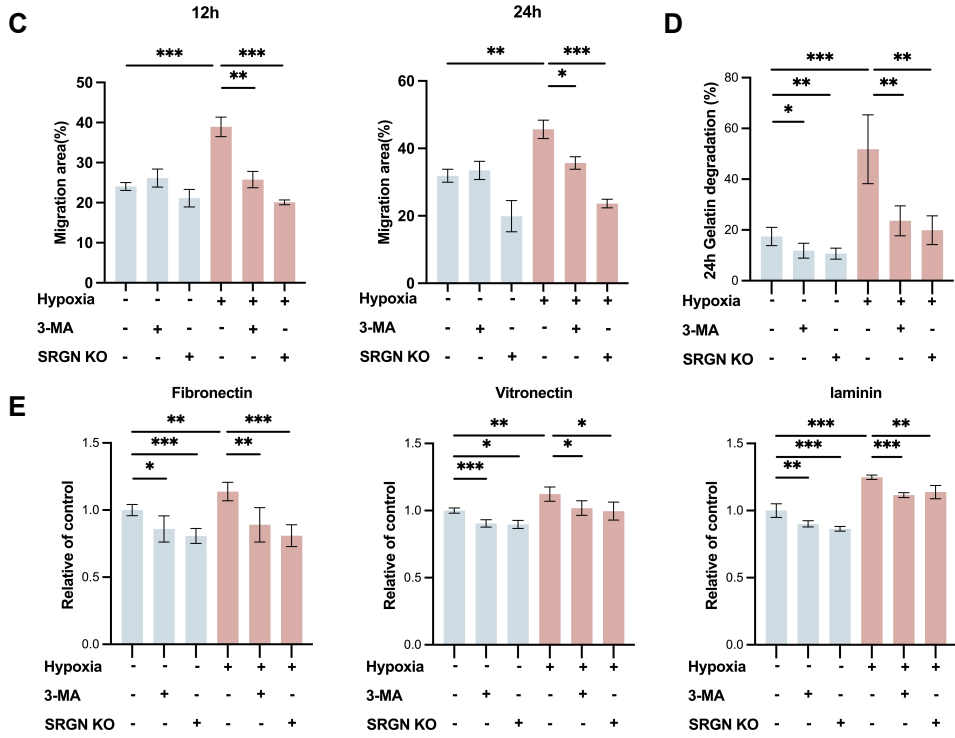

**A**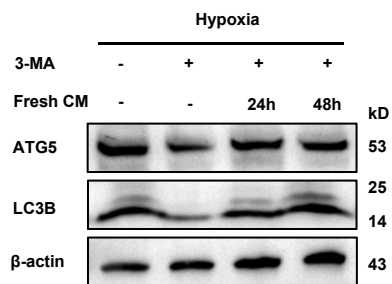**B**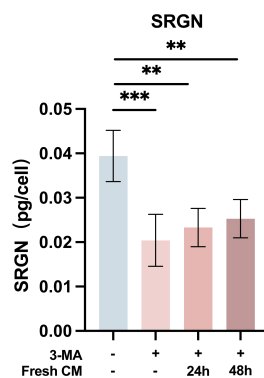

Supplement: Supplementary file 2 — Supporting information [file CTM2-15-e70556-s001.pdf]
